# CXCR6^+^ NK Cells in Human Fetal Liver and Spleen Possess Unique Phenotypic and Functional Capabilities

**DOI:** 10.3389/fimmu.2019.00469

**Published:** 2019-03-19

**Authors:** Laura S. Angelo, Lynn H. Bimler, Rana Nikzad, Kevin Aviles-Padilla, Silke Paust

**Affiliations:** ^1^Department of Pediatrics, Center for Human Immunobiology, Texas Children's Hospital, Houston, TX, United States; ^2^The Immunology Graduate Program at Baylor College of Medicine, Houston, TX, United States; ^3^Translational Biology and Molecular Medicine Graduate Program at Baylor College of Medicine, Houston, TX, United States; ^4^Department of Immunology and Microbiology, The Scripps Research Institute, La Jolla, CA, United States; ^5^The Integrative Molecular and Biomedical Sciences Graduate Program at Baylor College of Medicine, Houston, TX, United States

**Keywords:** natural killer cell, tissue residency, CXCR6, T-bet, Eomes, liver, spleen, innate immunity

## Abstract

Tissue-resident Natural Killer (NK) cells vary in phenotype according to tissue origin, but are typically CD56^bright^, CXCR6^+^, and CD69^+^. NK cells appear very early in fetal development, but little is known about when markers of tissue residency appear during gestation and whether the expression of these markers, most notably the chemokine receptor CXCR6, are associated with differences in functional capability. Using multi-parametric flow cytometry, we interrogated fetal liver and spleen NK cells for the expression of a multitude of extracellular markers associated with NK cell maturation, differentiation, and migration. We analyzed total NK cells from fetal liver and spleen and compared them to their adult liver and spleen counterparts, and peripheral blood (PB) NK. We found that fetal NK cells resemble each other and their adult counterparts more than PB NK. Maturity markers including CD16, CD57, and KIR are lower in fetal NK cells than PB, and markers associated with an immature phenotype are higher in fetal liver and spleen NK cells (NKG2A, CD94, and CD27). However, T-bet/EOMES transcription factor profiles are similar amongst fetal and adult liver and spleen NK cells (T-bet^−^/EOMES^+^) but differ from PB NK cells (T-bet^+^EOMES^−^). Further, donor-matched fetal liver and spleen NK cells share similar patterns of expression for most markers as a function of gestational age. We also performed functional studies including degranulation, cytotoxicity, and antibody-dependent cellular cytotoxicity (ADCC) assays. Fetal liver and spleen NK cells displayed limited cytotoxic effector function in chromium release assays but produced copious amounts of TNFα and IFNγ, and degranulated efficiently in response to stimulation with PMA/ionomycin. Further, CXCR6^+^ NK cells in fetal liver and spleen produce more cytokines and degranulate more robustly than their CXCR6^−^ counterparts, even though CXCR6^+^ NK cells in fetal liver and spleen possess an immature phenotype. Major differences between CXCR6^−^ and + NK cell subsets appear to occur later in development, as a distinct CXCR6^+^ NK cell phenotype is much more clearly defined in PB. In conclusion, fetal liver and spleen NK cells share similar phenotypes, resemble their adult counterparts, and already possess a distinct CXCR6^+^ NK cell population with discrete functional capabilities.

## Introduction

NK cells are immune cells that kill infected or malignant cells and secrete cytokines and chemokines (1). NK cells survey their environment through expression of activating or inhibitory cell surface receptors, as well as cytokine and chemokine receptors, and adhesion molecules (2, 3). NK cell expressed activating receptors, such as NKG2D (4–6) can be triggered by stress ligands expressed on infected or malignant cells. The cytotoxicity receptor CD16 binds Fc-domain of Immunoglobulin (Ig)G1 and directs NK cells killing toward antibody-coated targets (7), allowing crosstalk between the humoral and NK cell-mediated responses. However, healthy cells generally do not express sufficient amounts of stress ligands to activate NK cells, and further escape NK cell-mediated cytotoxicity by expressing healthy levels of self-Major Histocompatibility Complex Class I (MHC I) molecules that are ligands for NK cell-expressed inhibitory receptors (8).

Human NK cells are CD45^+^, CD56^+^ but CD3^−^ (9, 10), and can be identified using these markers by flow cytometry. In addition, intra-nuclear staining for the T-box transcription factors T-bet and Eomesodermin (EOMES) allow for the identification of specific NK cell developmental stages, and more recently, it has been appreciated that T-bet and EOMES are differentially expressed in tissue-specific NK cell subsets (11, 12). In human PB, 90% of all NK cells are considered mature NK cells that are highly cytotoxic, and identified phenotypically as T-bet^high^, EOMES^low^, CD56^low^, and CD16^high^. In contrast, the remaining ten percent of human PBMC-derived NK cells are T-bet^high^, EOMES^high^, CD56^high^, and CD16^low^ (13). CD56^high^ NK cells are generally considered less mature and less cytotoxic than CD56^low^ NK cells and are robust cytokine producers, and generally do not express much CD16. CD56^high^ and CD16^low^ NK cells however are abundant in human tissues, including human livers, spleens, and other secondary lymphoid organs (12, 14). Human adult spleen NK cells have been shown to express T-bet and/or EOMES, with EOMES more predominantly expressed in immature NK cells, while mature NK cells express higher levels of T-bet (13). Human adult liver also contains T-bet and/or EOMES expressing NK cells, as well as a subset of EOMES^high^ NK cells that co-express the G-protein coupled C-X-C Motif Chemokine Receptor 6 (CXCR6), and the activation or tissue residency marker CD69. In mice, CXCR6^+^ NK cells are largely confined to the liver and lung (15–17), while in humans, the majority of CXCR6^+^expressing NK cells are found in the liver, and to a lesser albeit significant extent in the spleen (17), bone marrow, and lymph nodes (18), with CXCR6^+^ NK cells largely absent from human PBMC (11, 14). The trans-membrane chemokine C-X-C motif ligand 16 (CXCL16) is the ligand for CXCR6, and is abundantly expressed on liver sinusoidal endothelial cells in mice and humans, facilitating the homing of CXCR6^+^ cells to those tissues (19), and providing survival signals to CXCR6^+^ immune cells (16, 17, 20, 21). Whether human non-liver tissues express significant amounts of human CXCL16 remains to be determined. Over the past decade, hepatic CXCR6^+^ NK cells were further demonstrated to mediate antigen-specific memory against haptens, virally encoded proteins, and viruses (16, 17, 22–24). Antigen-specific NK cell memory has also been described in rhesus macaques in both spleen and liver NK cells (25), albeit their CXCR6 expression was not investigated.

In the mouse it has been suggested that there are at least four populations of NK cells. These include conventional NK cells (located in the spleen and circulation), thymic NK cells, liver (tissue-resident) NK cells, and uterine NK cells (26, 27). Markers delineating human NK cell tissue residency have become a topic of interest in an effort to define specific tissue-resident NK cell phenotypes, which will distinguish these NK cells from their PB counterparts. As a result, several markers are strong candidates for delineating NK cell tissue residency, particularly in human liver, including CD69, CXCR6, CD49a, and CD49e (12, 14, 28–30).

While NK cells appear very early in fetal liver and spleen (6 and 15 weeks, respectively) (31), fetal NK cells, by PB NK cell standards, appear immature and functionally hypo-responsive. This may be prudent during gestation to avoid rejection of any maternal cells that might cross the maternal-fetal barrier in the placenta (32–34). However, fetal NK cells are capable of cytotoxic function, and can kill target cells naturally via the secretion of cytotoxic mediators and by antibody dependent cellular cytotoxicity (ADCC) and secrete large amounts of INFγ (32). It may therefore not be surprising that fetal NK cells are capable of degranulation, as evidenced by the upregulation of the lysosome-associated membrane glycoprotein CD107a in fetal liver, spleen, and lung NK cells in response to IL-12 and IL-18 (31, 32).

Recently, several investigators have described a liver-resident NK cell population high in CXCR6 and CD69 in adult human liver (11, 12, 14, 29). However, little is known about phenotypic and functional differences between splenic and hepatic NK cells, as well as fetal vs. adult NK cells, especially in the context of NK cell expressed CXCR6. Hence, we employed multi-parametric flow cytometry to discern phenotypic differences between CXCR6^−^ and CXCR6^+^ NK cells in fetal liver and spleen, including markers of NK cell differentiation and maturation as a function of gestational age, and we dissected the ability of fetal NK cell subsets to mediate cytotoxic effector functions despite their inherent immaturity, and compared our results to that of adult tissue matched NK cells.

We found that fetal liver and spleen NK cells resemble each other, as well as their adult counterparts more than they resemble PB NK cells. Fetal spleen and liver-derived NK cells are less mature in phenotype than adult PB-derived NK cells, as indicated by a lower expression of CD16, CD57, and KIR, and a higher expression of NKG2A, CD94, and CD27. However, T-bet/EOMES transcription factor profiles are tissue specific and similar amongst fetal and adult liver and spleen NK cells (T-bet^−^/EOMES^+^) but differ significantly from PB NK cells (T-bet^+^EOMES^−^). Further, donor-matched fetal liver and spleen NK cells share similar patterns of expression for most markers as a function of gestational age, and while fetal liver and spleen NK cells display limited cytotoxicity, both produced large amounts of TNFα and IFNγ and degranulated efficiently in response to stimulation with PMA/Ionomycin. Further, CXCR6^+^ NK cells in fetal liver and spleen produce more cytokines and degranulate more robustly than their CXCR6^−^ counterparts, even though CXCR6^+^ NK cells in fetal liver and spleen possess an immature phenotype. We conclude that major differences between CXCR6^−^ and CXCR6^+^ NK cell subsets appear to occur later in development, as a distinct CXCR6^+^ NK cell phenotype is much more clearly defined in PB. Our findings provide insight into the phenotypic identities and functional capabilities of human tissue-specific NK cells. These data may open the door for the development of tissue-specific clinical approaches and may inform new approaches in newborn or maternal vaccine development.

## Materials and Methods

### PBMCs From Healthy Donors

Healthy volunteers were used as the source of peripheral blood mononuclear cells (PBMCs) for all experiments. For PBMC isolation, peripheral blood was immediately diluted 1:1 with Ca^++^, Mg^++^ free phosphate buffered saline (PBS) and layered over Ficoll-Pacque (GE Healthcare), centrifuged at 2,000 rpm with no brake for 20 min at room temperature (RT). The lymphocyte layer was removed, washed, and counted. Multiparametric flow cytometry, degranulation, or ^51^Cr release assays were performed immediately.

### Fetal Liver and Spleen Tissue

Human fetal liver and spleen tissue was obtained from Advanced Bioscience Resources. Liver and spleen tissues were minced into fine pieces, then filtered through 40 μm filters before density centrifugation over Ficoll-Paque at 2,000 rpm for 25 min with no brake. Cells at the interface were harvested and washed with PBS and counted by trypan blue exclusion using a Cellometer to ensure uniform counting of all samples (Nexcelom). Fetal liver cells were depleted of CD34^+^ stem cells, which were used for the generation of humanized mice for an unrelated project, by positive selection (Miltenyi). The remainder/majority of the cells (column flow-through), along with fetal spleen cells were immediately used for flow cytometry, intracellular flow cytometry (ICFC), chromium release, or degranulation assays. None of the cells used for this study were frozen.

### Adult Liver and Spleen Tissue

Human adult liver perfusates were from cadaver donors, therefore informed patient consent was not required. 50–100 mL of liver perfusion was collected, centrifuged, and the cell pellet was resuspended in an appropriate volume of RPMI-1640 (HyClone) and separated by density centrifugation over Ficoll and handled as described above for blood samples. Cells were washed in PBS, counted, and used immediately for multiparameter flow cytometry. Adult spleen tissue was obtained from patients undergoing Pancreatic Ductal Adenocarcinoma (PDAC) surgery or splenectomy related to chronic pancreatitis. Patients had no known viral infections and had received no prior therapy. Informed consent was obtained from all patients undergoing PDAC surgery with splenectomy. The Internal Review Board of Baylor College of Medicine approved the study protocol and all studies were in accordance with the Declaration of Helsinki Principles. Adult spleen tissue was minced and passed through 40 μm filters, centrifuged and the cell pellet resuspended in RPMI. Lymphocytes were separated by density centrifugation over Ficoll and were used immediately for multiparameter flow cytometry. No frozen samples were used for this study.

### Cell Line Maintenance

For cytotoxicity and antibody dependent cytotoxicity assays (ADCC), K562 and Raji cell lines were purchased from ATCC and used as targets. All cell lines were maintained in RPMI-1640 + 10% FBS at 37°C, 5% CO_2_. Mycoplasma testing was routinely performed, and cell lines were confirmed to be negative.

### Multiparametric Flow Cytometry

In order to compare the phenotype of liver, spleen, and PB NK cells, we performed multiparametric flow cytometry. Lymphocytes harvested from fetal liver, fetal spleen, adult liver, adult spleen, and PB were freshly stained *ex vivo*. Cells were counted, and viability was assessed by trypan blue before staining. 10^6^ cells in 100 μL PBS were aliquoted into 5 mL FACS tubes. LIVE/DEAD fixable blue dead cell stain kit for UV excitation (ThermoFisher) was added for 7–10 min in the dark at RT. Extracellular antibodies were added for 20 min at RT (see Table S1 for all antibodies used in this study). For intranuclear staining of Tbet and EOMES, cells were washed with 2 mL of FACs buffer after extracellular antibodies, then resuspended in 1 mL of TONBO FOXp3/Transcription Factor Fix/Perm buffer (TONBO Biosciences) for 30 min at RT. After permeabilization, cells were washed with 2 mL of TONBO wash buffer. Intra-nuclear antibodies (anti-Tbet-BV711 and anti-EOMES-PerCP/Cy5.5) were added and incubated for 30 min at RT. Cells were washed with TONBO wash buffer, then FACS buffer and resuspended in a final concentration of 1% paraformaldehyde (PFA) and kept at 4°C in the dark until acquisition on a BD LSR Fortessa with 18 color configuration.

### Chromium Release Assays

NK cell cytotoxic function was evaluated by ^51^Cr-release assays using K562 erythroleukemia cells or Raji B lymphoma cells as targets for cytotoxicity and ADCC, respectively. Fresh bulk PBMCs from healthy donors were used as controls for both assays. The number of effector cells available determined the effector:target ratios for both assays. K562 or Raji target cells were labeled with 100 μCi ^51^Cr (Na_2_CrO_4_) (Perkin-Elmer) per 10^6^ cells for 1 h at 37°C and then washed 3 times with RPMI + 10% FBS (R10). For cytotoxicity assays, targets and effectors were either incubated in R10 media alone or in the presence of 1,000 IU/mL IL-2. For ADCC, 20 μg/mL of the anti-CD20 monoclonal antibody, Rituximab (Genentech) was added. Bulk effector and target cells were plated together into 96 well round bottom plates and incubated for 4 h at 37°C, 5% CO_2_. Following incubation, one row of targets was lysed with 1.0% IGEPAL (Sigma) for total release and plates were centrifuged at 300 × g for 10 min with no brake. Supernatants were harvested and transferred to a LUMA plate (Perkin Elmer) and dried overnight. Plates were counted on a TopCount NLT gamma detector (Perkin Elmer). Spontaneous release of ^51^Cr into the supernatant by target cells alone was used as background and subtracted from all experimental values. Percent specific lysis = (Experimental cpm – spontaneous release cpm)/Total cpm – spontaneous cpm) × 100.

### Degranulation Assays and Intracellular Flow Cytometry (ICFC)

The ability of NK cells to degranulate in response to K562 target cells at a 1:1 ratio was measured using CD107a expression (35, 36). Activating cytokines and other indices of NK cell cytolytic activity (TNFα, IFNγ, perforin, Granzyme B) were also measured using ICFC. One million cells were resuspended in R10 media alone as a negative control, PMA (1 μg/mL) + ionomycin (0.5 μg/mL) as a positive control, or K562 target cells (1:1 ratio) labeled with 2 μM CFSE (ThermoFisher Scientific). GolgiPlug (Brefeldin A) and GolgiStop (Monensin, BD Biosciences) were added to the tubes prior to incubation for 4 h at 37°C, 5% CO_2_. Anti-human CD107a-BV786 (BD Biosciences) was present during the entire incubation time. After stimulation, cells were washed with 2 mL of FACS buffer and stained with LIVE/DEAD fixable blue dead cell stain kit as described above. Extracellular antibodies were added for 20 min at RT. Cells were washed and permeabilized as described above. Intracellular antibodies (perforin-PE, Granzyme B-BV421, TNFα-PE/Cy7, and IFNγ-AF700) were added and incubated for 30 min at RT. Cells were washed and fixed in 1% PFA until acquisition. All staining steps were carried out in the dark. Samples were filtered prior to acquisition.

### Data Analysis and Software

All samples were acquired on a BD LSR Fortessa. FCS 3.0 files were transferred to FlowJo software Version 10.1 (TreeStar). The gating strategy is as shown in Figures S1–S5. Fluorescence minus one (FMO) stains were used in every experiment to determine positive and negative populations, and compensation was performed individually for each experiment using OneComp eBeads (eBioscience). Gates were applied from FMOs to samples, and values for mean fluorescence intensity (MFI) and percent positive cells were transferred to Graph Pad Prizm for statistical analysis.

### Statistical Analysis

The flow data are presented as individual data points with the mean and standard deviation indicated for each marker. Prism 7 (GraphPad) was used for all statistical analyses. For unpaired sample groups (liver vs. PB, etc.), Student's *t*-test with Welch's correction was employed. For paired samples (organs or NK cell subsets from the same individual), a paired Student's *t*-test was used. To compare 3 or more groups, a one-way ANOVA was performed followed by one of the following tests to adjust for multiple comparisons: a Dunnett's or Holm-Sidak's multiple comparison test to compare multiple groups to one control group, or a Tukey's correction to compare among all groups. Lastly, a two-way ANOVA was used to compare three or more groups within and between multiple conditions. *p* < 0.05 was considered statistically significant and designated as ^*^p < 0.05, ^**^p < 0.01, ^***^p < 0.001, and ^****^p < 0.0001.

## Results

### Hepatic and Splenic Fetal NK Cells Share a Similar Immature Tissue-Resident Phenotype

**Figure 1:**
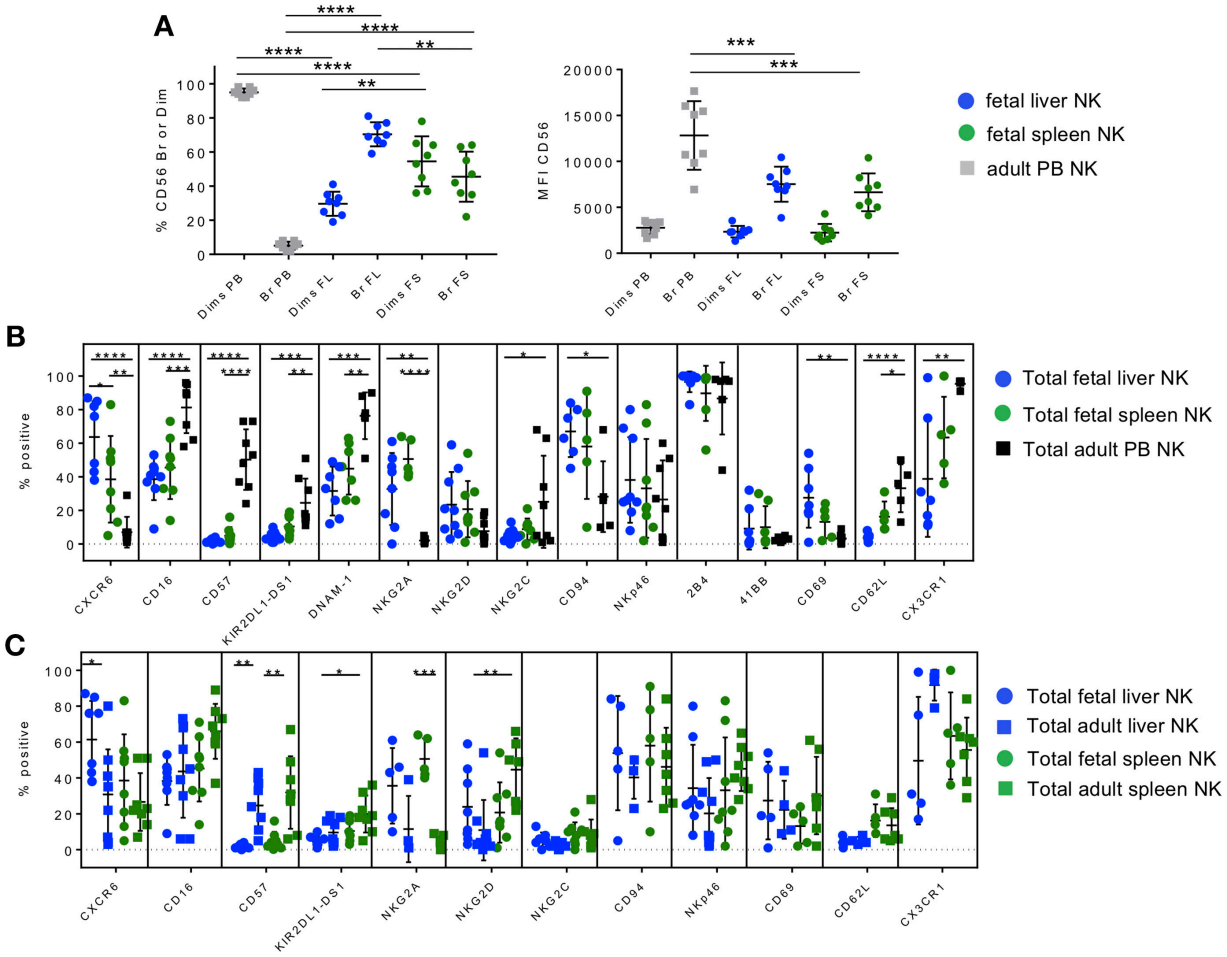
Hepatic and splenic fetal NK cells share a similar immature tissue-resident phenotype. Phenotypic profiling of fetal liver and spleen NK cell subsets. Gating strategy based on FMOs in all tissues is shown in Figures S1–S8. **(A)** Proportion of CD56^bright^ and CD56^dim^ NK cells (left) and CD56 mean fluorescence intensity (MFI) (right) in fetal liver (blue circles), spleen (green circles), and adult PB NK cells (gray squares). **(B)** Comparison of extracellular markers important in NK cell differentiation, maturation, and migration in fetal liver (blue circles), fetal spleen (green circles), and adult PB NK cells (black squares). **(C)** Adult liver and spleen NK cells are phenotypically similar and are more mature than fetal liver and spleen NK cells. Comparison of extracellular markers on fetal liver and spleen NK cells versus adult liver and spleen NK. Individual data points are shown. Bars represent mean and standard deviation for each marker. Bars represent mean and standard deviation for each marker. A one-way ANOVA with either Holm-Sidak's Multiple Comparison Test **(A)** or Tukey's Multiple Comparisons Test **(B,C)** was used to analyze unpaired samples (shown). Paired Student's *t*-tests were used to compare matched fetal tissue pairs where possible (see text). ^*^p < 0.05; ^**^p < 0.01; ^***^p < 0.001; ^****^p < 0.0001. *n* = 4–8.

To identify discrete phenotypic differences that distinguish fetal liver NK cells from fetal spleen NK cells, we used multi-parametric flow cytometry to interrogate multiple extracellular markers involved in NK cell differentiation and maturation in single cell suspensions of donor-matched fetal hepatic and splenic lymphocytes (Tables S1, S2). NK cells were defined as live cells that express CD45 and CD56 but not CD3, and flow cytometry data was gated as shown in Figures S2–S6. We found a much higher frequency of CD56^bright^ NK cells in fetal liver (70%) than fetal spleen (46%), and in fetal NK cell preparations compared to adult PB NK cells (5%) (Figures 1A, S2–S6). CD16 expression can be used to stratify CD56^bright^ and CD56^dim^ NK cells in PB since CD56^dim^ NK cells express much higher levels of CD16. We used a combination of CXCR6, CD16, and killer immunoglobulin receptors (KIR) (when expressed) to identify CD56^bright^ and dim subpopulations in fetal liver and spleen NK cells (32) (Figures S3, S4). Currently, CD56^dim^ NK cells are not considered tissue-resident in liver, but rather as non-resident NK cells passing through circulation (18, 19, 22, 37–40). Despite the high percentage of CD56^bright^ NK cells in fetal liver and spleen, the mean fluorescence intensity (MFI) for CD56 is actually significantly higher in the small population of CD56^bright^ NK cells from PB NK cells (Figure 1A, right panel). As expected, CD56^dim^ NK cells constitute a larger percentage of NK cells in the peripheral blood (mean 95%) and less of the NK cells in fetal liver (mean 30%) and spleen (mean 55%), while the MFI of CD56 does not differ significantly in the CD56^dim^ NK cells of all three tissues (Figure 1A, right panel). Fetal spleen NK cells contain a higher percentage of CD56^dim^ NK cells than fetal liver (*p* < 0.01), and thus a lower percentage of CD56^bright^ NK cells (*p* < 0.01). The high percentage of CD56^bright^ NK cells in fetal liver and spleen is consistent with the immature phenotype seen in adult tissue-resident NK cells (4, 18, 37, 41–43).

Using immuno-staining and flow cytometry, we compared the expression of a plethora of functionally relevant NK cell markers on fetal liver and spleen NK cells to adult PB NK cells. The chemokine receptor CXCR6 is highly expressed on adult human liver and spleen NK cells (14, 18, 37), while the ligand for CXCR6 (CXCL16) is highly expressed on liver sinusoidal epithelial cells and is believed to enhance survival and retention of this NK cell subset in the liver (17, 19). Fetal liver and spleen NK cells are also highly positive for CXCR6 (Figure 1B) compared to adult PB NK cells (*p* < 0.0001 and *p* < 0.01, respectively), with fetal liver NK cells expressing the highest level of CXCR6. Fetal liver NK cells express significantly lower levels of maturation markers than PB NK cells, including CD16, CD57, and KIR, which are involved in NK cell inhibition and education or “licensing” (44). This same pattern holds true for fetal spleen NK cells (Figure 1B). There is virtually no difference in maturation marker expression between fetal liver and fetal spleen NK cells, with the exception of KIR2DL1-DS1, which is higher in fetal spleen in a separate paired analysis (*p* < 0.01). This may be due to the presence of more CD56^dim^ NK cells in fetal spleen. DNAM-1 (DNAX Accessory Molecule-1), which is important in NK cell-mediated tumor immunity (45–48), is expressed on the surface of NK cells and is involved in NK cell activation by mediating adhesion to cells expressing its ligands (CD112 and CD155) (49, 50). DNAM-1 expression is high on the surface of PB NK cells compared to fetal liver (*p* < 0.001) and fetal spleen NK cells (*p* < 0.01) (Figure 1B). Representative flow plots for DNAM-1 are shown in Figure S7.

NKG2A, an inhibitory C-type lectin receptor, binds HLA-E on the surface of normal cells and provides an inhibitory signal to NK cells in combination with its partner CD94 (51). Both fetal liver and spleen NK cells express higher levels of NKG2A than peripheral blood NK cells, which is characteristic of immature NK cells and the immuno-tolerant environment thought to be present in both adult and fetal liver (Figure 1B). CD94 is more highly expressed in fetal liver than PB NK cells (*p* < 0.05), however, individual donors vary more in fetal spleen NK cells and is not statistically different from PB NK cells. This is not unexpected since CD94 is lost as NK cells mature and is frequently higher in the CD56^bright^ NK cell subset in PB and lymphoid tissue (18, 51, 52). NKG2D, an important NK cell activating receptor, is not significantly different among the three tissues. NKG2C is present in a subset of expanded NK cells following infection with cytomegalovirus (CMV), and is typically expressed at low levels unless infection has occurred (53). One set of fetal tissue may have been positive for CMV infection based on elevated NKG2C (13% in fetal liver and 21% in fetal spleen NK cells), although CMV status is unknown (Figure 1B). One would expect levels of NKG2C to be much lower in fetal NK cells than PB NK cells due to lack of CMV experience. Interestingly, levels of NKG2C are slightly higher in fetal spleen NK cells than donor-matched fetal liver NK cells in the paired analysis (*p* < 0.05, not shown), while fetal liver NKG2C expression is significantly lower than PB NK cells (*p* < 0.05) (Figure 1B). The activation marker and natural cytotoxicity receptor NKp46 is not significantly differentially expressed in fetal liver or spleen NK cells compared to PB NK cells. This is somewhat surprising in that NKp46 is typically expressed at higher levels in CD56^bright^ NK cells (18). We interrogated two additional NK cell activation markers, 2B4 and 41BB. Representative flow plots for 2B4 and 41BB are shown in Figure S7. 2B4 is a cell surface receptor on NK cells that is thought to mediate non-MHC restricted killing and can modulate NK cell activity when binding its ligand CD48 on neighboring cells (54, 55). 2B4 is highly expressed on the surface of NK cells from all three tissues (Figure 1B). 41BB is typically present only upon activation of NK cells (56). Hence, as expected 41BB is virtually absent from all NK cells studied (Figure 1B). The early activation/tissue residency marker CD69 is highly expressed in adult human liver NK cells (14, 18) and is also more abundant on fetal liver than PB NK cells (*p* < 0.01).

Two markers of lymphocyte migration, CD62L (L-selectin) and CX3CR1 (fractalkine receptor), are expressed at lower levels in fetal liver and spleen NK cells compared to PB NK cells. Lower expression of these migration markers on fetal NK cells is consistent with their presumed role as tissue-resident cells that do not frequently migrate to other areas of the body. Taken together fetal liver and spleen NK cells are phenotypically immature compared to PB NK cells but are similar to each other in most markers studied, with the exception of a few markers of differentiation/maturity that are more highly expressed on fetal spleen NK cells (KIR, NKG2C, and CD62L).

### Adult Liver and Spleen NK Cells Are Phenotypically Similar, and Are More Mature Than Fetal Liver and Spleen NK Cells

After comparing the phenotypes of fetal liver and spleen NK cells to PB NK cells, we were curious how similar fetal liver and spleen NK cells are to their adult counterparts. We performed the same phenotypic analysis with adult liver and spleen NK cells and compared them to fetal liver and spleen NK cells. This level of phenotyping comparing fetal and adult liver and spleen NK cells has not been executed in detail to the best of our knowledge. CXCR6 is more abundantly expressed on adult liver and spleen NK cells than PB [(14, 18, 19, 29, 37) and compare Figures 1B,C]. The *p*-values in Figure 1C pertain to the relationship of fetal to adult NK cells, and the relationship of adult liver to adult spleen NK cells since fetal liver and spleen NK cell comparisons were performed in Figure 1B. CXCR6 is more highly expressed on fetal liver NK cells compared with adult liver NK cells (*p* < 0.05) (Figure 1C). In terms of maturity markers, a much higher percentage of adult NK cells express CD57 compared to fetal NK cells (*p* < 0.01), but CD57 is expressed equally in adult liver and spleen. Adult spleen NK cells express higher levels of KIR2DL1-DS1 than adult liver NK cells (*p* < 0.05), indicating that spleen NK cells may be more “highly educated” than liver NK cells. CD16 expression trends higher in adult spleen NK cells compared to fetal spleen NK cells and adult liver NK cells but is not statistically significant (Figure 1C), while NKG2A levels are much higher in fetal spleen NK cells than in their adult counterparts, which is consistent with their less mature stage of development (*p* < 0.001).

In terms of activating receptors, NKG2D is significantly higher in adult spleen NK cells compared to adult liver (*p* < 0.01), suggesting that adult spleen NK cells are more highly activated. There is no significant difference in NKp46 expression among all four tissues, although adult spleen NK cells have the highest mean (45%), again suggesting activation. NKG2C is not statistically different in adult liver and spleen NK cells, however, these are not donor-matched samples so CMV status, and hence NKG2C expression, varies amongst donors. CD94 is not significantly different in any comparison, while CD69 does not differ significantly between liver and spleen NK cells (fetal or adult), which indicates that CD69 is present early in liver and spleen development (see Figure S11C). There is no differential expression of CD62L or CX3CR1. DNAM-1, 2B4, and 41BB were not analyzed for adult tissues. Taken together, fetal liver and spleen NK cells strongly resemble each other phenotypically, as do adult liver and spleen NK cells. Adult NK cells express higher mean percentages of maturation markers including CD16, CD57, and KIR, although not all of these differences are statistically significant, while fetal NK cells express more NKG2A. Adult spleen NK cells appear to have a more activated phenotype based on increased mean expression of NKG2D and NKp46.

### CXCR6^−^ and CXCR6^+^ NK Cells Show Phenotypic Differences During Development

**Figure 2:**
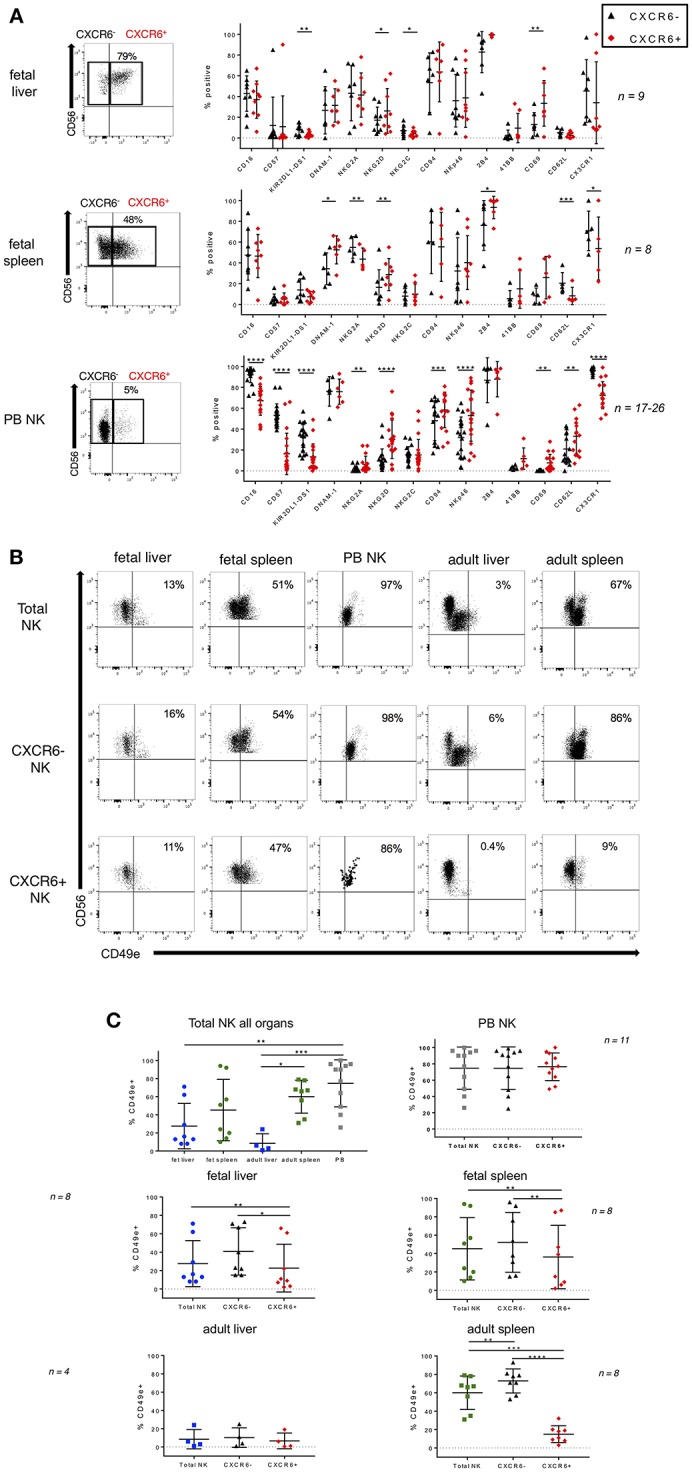
Phenotypic differences in CXCR6^−^ and CXCR6^+^ NK cell subsets in fetal liver and spleen. **(A)** Representative dot plots for gating CXCR6^−^ and + NK cells are shown. Comparison of NK cell markers on CXCR6^−^ (black) and CXCR6^+^ (red) NK cells in fetal liver, spleen, and adult PB NK cells (*n* = 8–26). **(B)** Representative flow plots showing expression of CD49e in fetal liver and spleen, adult liver and spleen, and adult PB NK cells in total, CXCR6^−^, and CXCR6^+^ NK cell subsets. **(C)** CXCR6^+^ NK cells express lower levels of CD49e than CXCR6^−^ NK cells in fetal liver and spleen. Individual data points showing CD49e expression on total, CXCR6^−^, and CXCR6^+^ NK cell subsets in fetal liver (blue circles) and spleen (green circles), adult liver (blue squares) and spleen (green squares), and adult PB NK cells (gray squares) (*n* = 4–11). Bars represent mean and standard deviation for each marker. A one-way ANOVA with Tukey's Multiple Comparisons Test was performed for unpaired samples and a Student's *t*-test for paired CXCR6^−^ and + NK cells within the same donor. *P*-values are as described in the legend to Figure 1.

CXCR6 is highly expressed on fetal liver and spleen NK cells as early as 17 weeks gestation (Figures 1C, S11A). Little is known about the specific phenotype and functional capabilities of CXCR6^+^ NK cells during development. Since CXCR6^+^ liver NK cells have been implicated in NK cell memory to haptens and viral antigens in mice (17), a thorough understanding of the phenotype and function of this NK cell subset may inform maternal/fetal vaccine development. We therefore examined phenotypic differences in terms of differentiation and maturation markers between CXCR6^−^ and CXCR6^+^ NK cell subsets in fetal liver and spleen and for comparison, adult PB NK cells. CD16 and CD57 are expressed equally in both CXCR6^−^ and CXCR6^+^ fetal liver and spleen NK cells, however, KIR2DL1-DS1 is expressed at lower levels on CXCR6^+^ fetal liver NK cells than on their CXCR6^−^ counterparts (*p* < 0.01). In contrast, CXCR6^+^ PB NK cells express less CD16, CD57, and KIR2DL1-DS1 than CXCR6^−^ PB NK cells, consistent with a more immature, adult “liver-like” phenotype. DNAM-1 is expressed equally in CXCR6^−^ and CXCR6^+^ NK cells of PB and fetal liver, while CXCR6^+^ NK cells in fetal spleen express higher levels of DNAM-1 (*p* < 0.05). This is noteworthy because DNAM-1^+^ NK cells produce more cytokines and display enhanced IL-15 signaling (50), which is consistent with the cytokine-secreting role previously published for CXCR6^+^ NK cells (14). NKG2D is more highly expressed on CXCR6^+^ NK cells in fetal liver and spleen (*p* < 0.05 and *p* < 0.01, respectively) (Figure 2A). NKG2A is typically associated with an immature phenotype and is surprisingly more highly expressed on CXCR6^−^ NK cells in fetal spleen (*p* < 0.01) but is higher in CXCR6^+^ NK cells in PB. As mentioned previously, one set of fetal tissue appears positive for CMV infection, which accounts for the increased NKG2C expression observed in one of the pairs (Figure 1B). In fetal liver, NKG2C is lower in CXCR6^+^ NK cells and is more highly associated with CXCR6^−^ NK cells, but this is not the case with this specific set of donors for PB and fetal spleen NK cells (Figure 2A). Activation markers including NKp46, 2B4, 41BB, and CD69 are typically more highly expressed on CXCR6^+^ NK cells, but this is not the case for all markers and tissues (Figure 2). CXCR6^+^, CD69^+^ NK cells have been characterized as liver/tissue-resident, while CXCR6^−^ NK cells are considered non-resident and express less CD69. We assessed differences in the ability of CXCR6^−^ and + NK cell subsets to migrate in the three tissues. Homing receptor expression (CD62L) indicates that CXCR6^+^ NK cells in PB are capable of migrating to lymph nodes or sites of inflammation at various locations throughout the body, whereas fetal CXCR6^+^ NK cells maintain a very low expression of CD62L, indicating they do not migrate at this point in development (Figure 2A). CX3CR1, however, is lower on CXCR6^+^ NK cells in PB and fetal spleen, suggesting that migration of CXCR6^+^ NK cells may be more complicated than merely CD62L expression levels (Figure 2A). Taken together, CXCR6^+^ NK cells in fetal liver and spleen differ from CXCR6^−^ NK cells in KIR, activation, and migration receptors, and express more CD69. Importantly, CXCR6^+^ NK cells in PB most closely resemble the published phenotype of CXCR6^+^ NK cells in the liver.

### CXCR6^+^ NK Cells Express Lower Levels of CD49e Than CXCR6^−^ NK Cells in Fetal Liver and Spleen

Lack of CD49e was recently shown to be a robust marker of tissue resident NK cells in adult human liver (30). The source of adult liver NK cells used in that study was the final post-excision liver flush (30). CD49e (integrin alpha 5), when paired with beta 1 integrin forms VLA-5, otherwise known as fibronectin receptor. We wanted to determine if CD49e is also absent from fetal liver NK cells. The source of our fetal liver NK cells is whole tissue, not perfusate, so some variability is to be expected. We also obtained several adult liver perfusates for comparison purposes. We examined CD49e expression in total, CXCR6^−^, and CXCR6^+^ NK cells from fetal liver, fetal spleen, adult liver, adult spleen, and adult PB. Representative flow plots are shown in Figure 2B. When comparing total NK cells among all tissues, liver NK cells express the lowest levels of CD49e, which is consistent with currently published data. Adult spleen NK cells express more CD49e than adult liver NK cells (*p* < 0.05), and PB NK cells express the highest levels. CD49e is not differentially expressed on fetal liver and spleen NK cells (Figure 2C, upper left). CXCR6^+^ NK cells express lower levels of CD49e than their CXCR6^−^ counterparts in all tissues, except for adult liver, where the expression levels are low in both subsets and peripheral blood where the expression levels are high in both subsets. Adult spleen NK cells have much lower levels of CD49e expression in the CXCR6^+^ subset than in the CXCR6^−^ subset. The difference is striking (*p* < 0.0001). In contrast, peripheral blood NK cells express CD49e at very high levels in total, CXCR6^−^, and CXCR6^+^ subsets with no significant difference between subsets (Figure 2C, upper right). It appears that CD49e expression starts at higher levels in fetal liver and spleen CXCR6^+^ NK cells and decreases during development (Figure S11C). Loss of CD49e in adult CXCR6^+^ spleen NK cells may indicate acquisition of a tissue-resident phenotype.

### Tbet and EOMES Transcription Factor Profiles Show Distinct Patterns in CXCR6^−^ and CXCR6^+^ NK Cell Subsets

**Figure 3:**
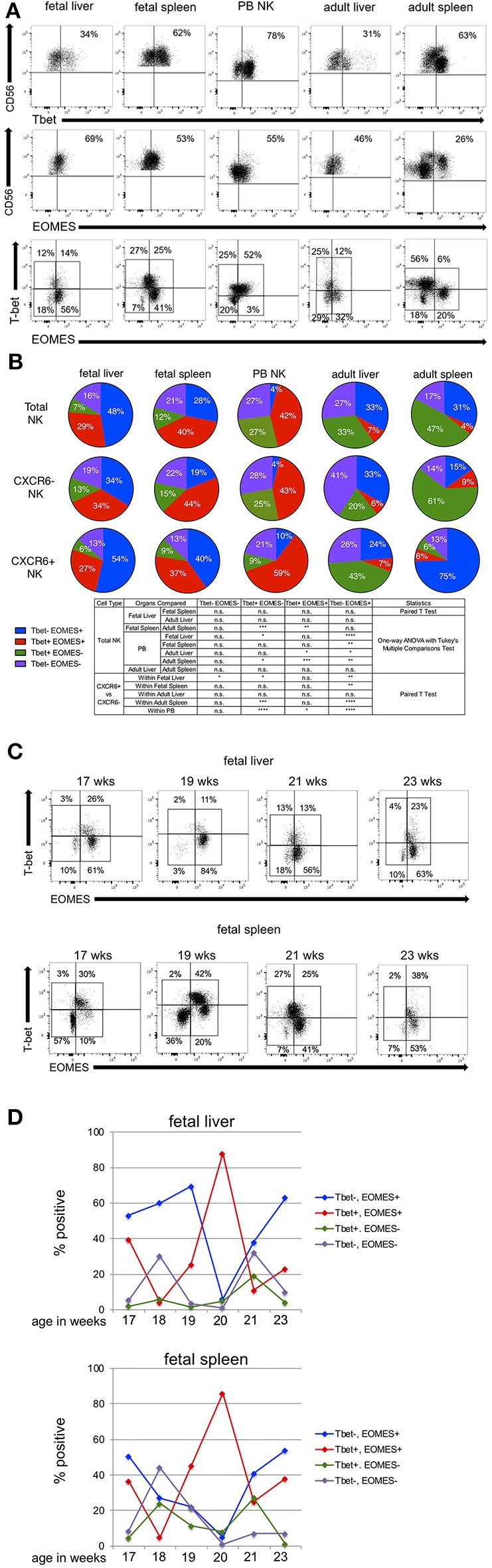
Tbet and EOMES transcription factor profiles show distinct patterns in CXCR6^−^ and CXCR6^+^ NK cell subsets. **(A)** Representative flow plots showing gating strategy for Tbet, EOMES, and Tbet/EOMES subsets in total NK cells (see Figure S9 for FMOs). **(B)** Expression of Tbet/EOMES subsets in total, CXCR6^−^ and CXCR6^+^ fetal liver (*n* = 8), fetal spleen (*n* = 8), PB (*n* = 18), adult liver (*n* = 3), and adult spleen (*n* = 7) NK cells. Total, CXCR6^−^, or CXCR6^+^ NK cells were gated according to FMOs and percentages of four subsets of Tbet/EOMES-expressing NK cells were quantified as parts of a whole: Tbet^−^EOMES^−^ (purple); Tbet^−^EOMES^+^ (blue); Tbet^+^EOMES^+^ (red); and Tbet^+^EOMES^−^ (green). Donor-matched fetal liver and spleen were compared using Student's *t*-test, as were CXCR6^−^ and CXCR6^+^ NK cells within the same donor. Unmatched adult liver and spleen NK cell subsets were compared to fetal liver and spleen NK cells and peripheral blood NK cells using a one-way ANOVA with Tukey's Multiple Comparisons Test (see Table). **(C)** Representative flow plots for Tbet/EOMES expression in fetal liver and spleen NK cells analyzed by gestational age. **(D)** Summary of the expression of Tbet/EOMES-expressing NK cell subsets in fetal liver and spleen as a function of gestational age. The majority of time points are represented by one sample (*n* = 1), with the exception of weeks 17 and 19 (*n* = 2). Tbet^−^EOMES^−^ (purple line); Tbet^−^EOMES^+^ (blue line); Tbet^+^EOMES^+^ (red line); Tbet^+^EOMES^−^ (green line).

Tbet and EOMES are both members of the T box family of transcription factors and are important in the differentiation and effector functions of both T and NK cells (13, 57–59). Differential expression of Tbet and EOMES is associated with different stages of NK cell development and differentiation. Peripheral blood NK cells habitually express low levels of Tbet^−^EOMES^+^ and higher levels of Tbet^+^EOMES^−^ subsets. Expression of Tbet is associated with cytotoxic effector function, whereas EOMES is correlated with the ability to produce cytokines in human NK cells (13, 57, 58). Using intra-nuclear flow cytometry, we examined Tbet/EOMES expression in fetal and adult liver and spleen NK cells. Representative flow plots are shown in Figure 3A, and FMO controls are in Figure S9. Statistical analysis comparing Tbet/EOMES expression in NK cells between fetal and adult tissues and within tissues (CXCR6^−^ vs. CXCR6^+^) is detailed in the table below Figure 3B. In total PB NK cells, we see a large population of Tbet^+^EOMES^+^ (red) followed by a robust proportion of Tbet^+^EOMES^−^ NK cells (green) (Figure 3B). CXCR6^−^ NK cells in PB very closely resemble the total NK cell population, most likely because CXCR6^−^ NK cells make up the majority of peripheral blood NK cells (Figure 3B). CXCR6^+^ NK cells in the periphery differ significantly from their CXCR6^−^ counterparts by having a greater percentage of Tbet^+^EOMES^+^ (red) and lower Tbet^+^EOMES^−^ (green), consistent with their more classically immature, cytokine-secreting phenotype. In total fetal liver NK cells, there is a higher proportion of Tbet^−^EOMES^+^ cells (blue) than in PB (*p* < 0.0001). The Tbet^+^EOMES^+^ NK cell subset is also highly prevalent in total fetal liver NK cells, but there are very few mature Tbet^+^EOMES^−^ NK cells. In fetal liver, the majority of the NK cells are CXCR6^+^ hence the CXCR6^+^ NK cells in this tissue resemble the total NK cell population. CXCR6^−^ NK cells in fetal liver express a lower percentage of Tbet^−^EOMES^+^ than CXCR6^+^ NK cells (*p* < 0.01) (Figure 3B). More mature Tbet^+^EOMES^−^ NK cells (green) are low throughout fetal liver and spleen. Hence, fetal liver and spleen NK cells are similar in their Tbet/EOMES profiles. CXCR6^−^ fetal spleen NK cells contain a larger proportion of Tbet^+^EOMES^+^ like the total fetal spleen NK cell population, since they make up the majority of NK cells in this tissue. Tbet^−^EOMES^−^ (purple) NK cells do not vary greatly between subsets in each tissue with the exception of fetal liver (*p* < 0.05) (Figure 3B).

We next wanted to examine alterations in Tbet/EOMES transcription factor profiles between fetal NK cells and their adult counterparts. Comparing total fetal liver NK cells to adult liver NK cells, there is a switch to a more mature Tbet^+^EOMES^−^ profile in adult liver, and fewer Tbet^+^EOMES^+^, however, these differences are not statistically significant, perhaps due to the low number of adult liver perfusions we were able to obtain for intra-nuclear staining. CXCR6^+^ adult liver NK cells have a higher proportion of Tbet^+^EOMES^−^ than their CXCR6^−^ counterparts (Figure 3B). While this is unexpected based on prior publications, these differences are not statistically significant. Adult liver and spleen NK cells look similar in terms of total NK cells; however, adult spleen NK cells contain more Tbet^+^EOMES^−^ than adult liver (*p* < 0.05), again demonstrating a more mature phenotypic profile. Total fetal spleen NK cells are very different from adult spleen NK cells in that fetal spleen express more Tbet^+^EOMES^+^ NK cells (*p* < 0.01), while adult spleen exhibits a more mature Tbet^+^EOMES^−^ profile (*p* < 0.001). Interestingly, CXCR6^−^ NK cells in adult spleen are very high in Tbet^+^EOMES^−^ and lower in EOMES expressing NK cells (either Tbet^−^EOMES^+^ or Tbet^+^EOMES^+^) and resemble total adult spleen NK cells (Figure 3B). Overall, CXCR6^+^ NK cells typically exhibit a more EOMES^+^ phenotype when compared to their CXCR6^−^ counterparts.

Tbet/EOMES expression appears early in NK cell development, and EOMES expression is crucial in defining the NK cell lineage apart from other Innate Lymphoid Cells (ILC)1s (38, 58). We analyzed Tbet/EOMES expression in fetal liver and spleen NK cells as a function of gestational age to discern unique patterns of expression between the two organs. Since fetal tissue is difficult to acquire, we have only one sample for each gestational age, with the exception of 17 and 19 weeks where *n* = 2 or 3. Representative dot plots showing gating for Tbet/EOMES expression in fetal liver and spleen at four different gestational ages separated by 2 week intervals are shown in Figure 3C. FMOs for Tbet/EOMES gating are shown in Figure S9. Although each time point is from a different donor, the Tbet/EOMES transcription factor profiles can be compared between donor-matched fetal liver and spleen (Figure 3D). Similarities in the expression of Tbet/EOMES NK cell subsets in fetal liver and spleen NK cells as a function of gestational age are clearly apparent. Changes in Tbet^+^EOMES^+^ NK cells as a function of gestational age are virtually identical between fetal liver and spleen (red lines, Figure 3D), although the significance of the peak in this subset at 20 weeks in both tissues is unclear. We examined the expression of additional transcription factors including RORγt and GATA3 but saw little to no expression of these transcription factors in fetal liver and spleen NK cells according to our gating strategy. Hence, we could not distinguish any ILCs in the NK cells populations we studied (38).

### CXCR6^+^ NK Cells Exhibit a Less Mature Phenotype in Terms of CD27, CD11b

**Figure 4:**
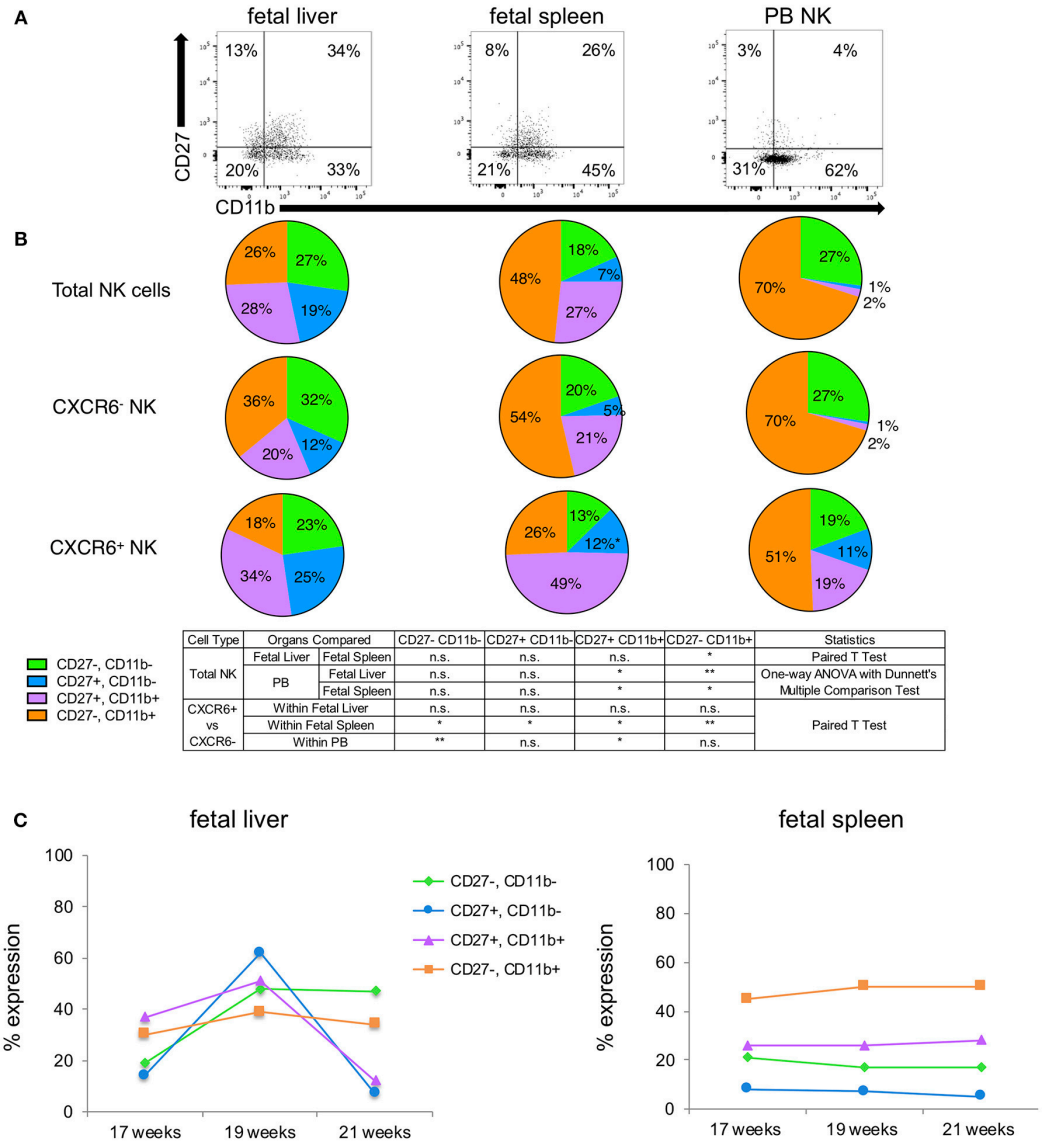
CXCR6^+^ NK cells exhibit a less mature phenotype in terms of CD27, CD11b. **(A)** Representative flow plots showing expression of four subsets of CD27 and CD11b-expressing NK cells within total NK cells of fetal liver, spleen, and PB (*n* = 3). FMOs are in Figure S10. **(B**) CD27, CD11b-expressing NK cells were quantified as parts of a whole as follows: CD27^−^CD11b^−^ (green); CD27^+^CD11b^−^ (blue); CD27^+^CD11b^+^ (purple); CD27^−^CD11b^+^ (orange). Statistics were as described in the table. **(C)** Expression of CD27, CD11b subsets in total fetal liver and spleen NK cells as a function of gestational age. Colored lines correspond to subpopulations as described in **(B)** (*n* = 1 for each time point).

CD27 and CD11b have frequently been used to ascertain murine NK cell developmental stages but are not widely used in this capacity for determining human NK cell developmental stage. Despite this, it is widely accepted that immature human NK cells are CD27^+^CD11b^−^ and mature human NK cells are CD27^−^CD11b^+^ [(60–64) and PB NK cells in Figure 4A]. Four subsets can be identified in order of maturity: CD27^−^CD11b^−^, CD27^+^CD11b^−^, CD27^+^CD11b^+^, and CD27^−^CD11b^+^. Representative flow plots for these subsets in fetal liver and spleen and PB NK cells are shown in Figure 4A and FMO overlays are in Figure S10. Total fetal liver NK cells express almost equal percentages of the four subsets, however, CXCR6^−^ fetal liver NK cells appear more mature than CXCR6^+^ fetal liver NK cells based on the prevalence of CD27^−^CD11b^+^ NK cells (orange wedge), but these differences are not statistically significant (Figure 4B). CD27^−^CD11b^+^ mature NK cells are thought to have higher cytolytic function (60, 65). Looking at the total percentage of CD27^+^ cells (CD27^+^CD11b^−^ plus CD27^+^CD11b^+^), CXCR6^+^ NK cells are less mature (blue plus purple wedges). CD27 single and double positive NK cells are associated with higher cytokine secretion in human decidua (60, 65). Total fetal spleen NK cells exhibit a more mature phenotype than total fetal liver NK cells (*p* < 0.05) (orange). CXCR6^−^ NK cells in fetal spleen closely resemble the total fetal spleen NK population. CXCR6^+^ NK cells in fetal spleen are less mature based on the low expression of CD27^−^CD11b^+^ (orange wedge) and high expression of CD27 (blue plus purple wedges) (Figure 4B). Peripheral blood NK cells are the most mature NK cells as expected, with 70% CD27^−^CD11b^+^ (fetal liver *p* < 0.01; fetal spleen *p* < 0.05). CD27 is extremely low in PB NK cells. CXCR6^−^ NK cells in PB are identical in CD27/CD11b subsets to total PB NK cells, again because CXCR6^−^ NK cells make up the majority of NK cells in the peripheral blood. CXCR6^+^ PB NK cells have a less mature phenotype [51% CD27^−^CD11b^+^ (orange), 30% CD27^+^ (blue + purple)] (Figure 4B). This is the same pattern seen with Tbet, EOMES, and extracellular markers; namely that CXCR6^+^ NK cells exhibit an immature phenotype in all tissues examined based on the expression of numerous markers. We were curious to see if there were any trends in the expression of CD27 and CD11b as a function of gestational age. We were severely limited by the number of samples we were able to phenotype; thus, we have one sample for each gestational age, but fetal liver and spleen can be directly compared as they are from the same donor. We found that fetal liver NK cells are much more dynamic in their expression of CD27, CD11b subsets (Figure 4C), while all four subsets remain constant in fetal spleen. Fetal spleen NK cells maintain a higher frequency of CD27^−^ CD11b^+^ and a lower frequency of the more immature subsets (green, blue, and purple lines) at all three gestational ages. The frequency of CD27, CD11b subsets in fetal liver NK cells from the same donor are much more dynamic, with fluctuations in the expression of 3 out of the 4 subsets. The exception is the more mature CD27^−^ CD11b^+^ NK cells, which is the one subset that remains constant (Figure 4C).

### Fetal Liver and Spleen NK Cells Show Remarkably Similar Patterns of Expression As a Function of Gestational Age

Although it is extremely difficult to track the appearance of specific NK cell markers over time during development in human fetal liver and spleen NK cells, we compared the expression of NK cell markers in donor-matched fetal liver and spleen as a function of gestational age (Figure S11). Lines are average expression. Dots represent individual data points. CXCR6 expression diverges in fetal liver and spleen, starting higher in fetal liver NK cells and gradually decreasing until 20 weeks where it holds fairly steady, ending at 40% CXCR6^+^. Fetal spleen NK cells take the opposite course, starting low and ending at 80% CXCR6^+^ at 23 weeks (Figure S11A). Maturation markers CD57 and KIR2DL1-DS1 remain very low on NK cells from both fetal liver and spleen for all gestational ages except for two samples, which may be outliers or contamination with maternal NK cells. CD16 expression is mirrored closely in fetal liver and spleen NK cells, starting at almost the same level of expression, with fetal spleen NK cells ending much higher than fetal liver NK cells (76 vs. 39%) (Figure S11A).

We next examined the expression pattern of NKG2A as a function of gestational age and found that NKG2A is more highly expressed in fetal spleen NK cells at all gestational ages examined (Figure S11B). Overall, NKG2A expression levels do not differ significantly between fetal liver and spleen NK cells when the data is taken together regardless of gestational age (Figure 1B). NKG2D follows a strikingly similar pattern of expression in fetal liver and spleen NK cells, with only slight differences in the percent positive NK cells at 17 weeks (Figure S11B). CD94 expression is also similar in fetal liver and spleen NK cells with the exception of 17 and 20 weeks. The overall impression is that the expression patterns are somewhat different in the two organs for CD94, which is known to decrease as NK cells mature (52). Interestingly, the expression pattern of NKp46 is strikingly similar between fetal liver and spleen NK cells (Figure S11B).

Adult liver NK cells lack expression of CD49e, so it is notable that overall CD49e expression is lower in fetal liver NK cells than in donor-matched fetal spleen NK cells as a function of gestational age (Figure S11C). Furthermore, expression of CD49e gradually decreases with increased gestational age in fetal liver NK cells. Overall, the expression pattern of CD69 looks similar in fetal liver and spleen cells. L-selectin (CD62L) is barely present on fetal liver NK cells, indicating that these cells are too immature to enter high endothelial venules. Fetal spleen NK cells do express CD62L at the earlier gestational ages, but it is very low by weeks 20 and 21. The pooled data show that CD62L is expressed at significantly higher levels on fetal spleen NK cells (Figure 1B). CX3CR1, also involved in lymphocyte migration, is more highly expressed in fetal spleen NK cells at any given gestational age, but the overall pattern of expression is similar to that of fetal liver NK cells (Figure S11C). Higher expression of CX3CR1 in fetal spleen NK cells did not reach statistical significance in the paired analysis (Figure 1B).

### Fetal Liver and Spleen NK Cells Produce IFNγ and TNFα Following Stimulation With PMA/Ionomycin

**Figure 5:**
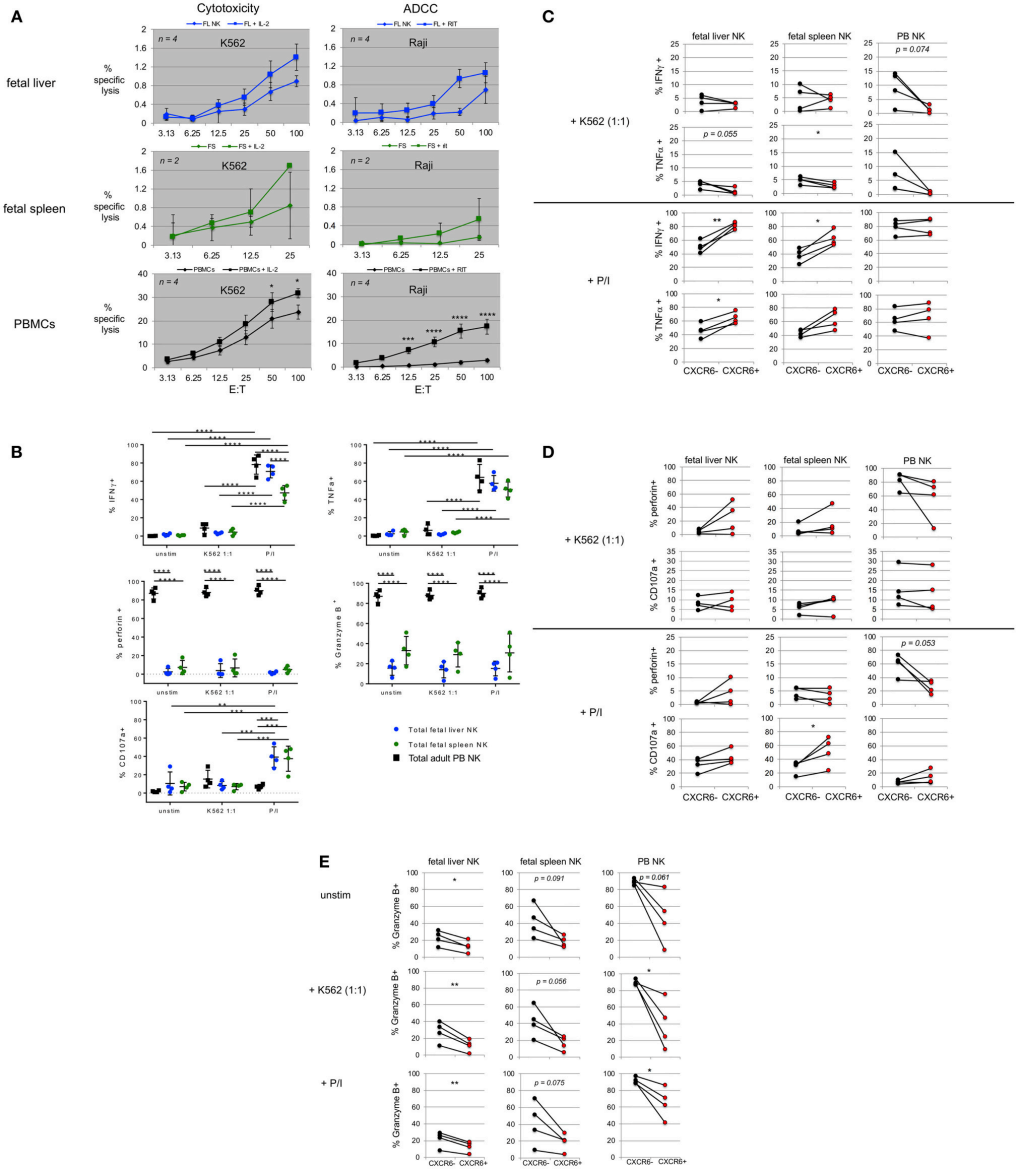
Fetal liver and spleen NK cells have low killing capacity but produce cytokines and degranulate in response to stimulation. **(A)** Bulk fetal liver (*n* = 4) and spleen (*n* = 2) lymphocytes were plated at various effector to target cell ratios (E:T) with ^51^Cr-labeled target cells. K562 were used as targets for cytotoxicity assays in the presence or absence of IL-2. Raji B lymphoma cells were used as targets for ADCC, plus or minus rituximab. PBMCs from healthy donors were used as controls. **(B)** Intracellular flow cytometry (ICFC) was used to assess fetal liver and spleen NK cell function. Total NK cells were gated for markers of cytotoxic function including production of IFNγ, TNFα, perforin, Granzyme B, and CD107a (degranulation). Total NK cells were stimulated with CFSE-labeled K562 target cells at a 1:1 ratio or PMA/ionomycin (P/I). Unstimulated cells (unstim) were incubated in media alone. Incubation was for 4 h. Fetal liver (blue circles), fetal spleen (green circles), and PB NK cells (black squares) (*n* = 4). **(C–E)** CXCR6^+^ fetal liver and spleen NK cells possess a unique functional profile with respect to mediators of NK cell effector function. Differences in the contribution of CXCR6^−^ NK cells (black circles) and CXCR6^+^ NK cells (red circles) in the production of IFNγ, and TNFα, **(C)** perforin, and CD107a **(D)** and Granzyme B, **(E)** in fetal liver and spleen NK cells following stimulation. PB NK cells were used as controls. Matched pairs were analyzed for statistical significance using the Student's *t*-test. *P*-values are as shown or as detailed in the legend to Figure 1.

Although it is known that fetal NK cells are immature and do not possess the same ability to specifically lyse target cells as PB NK cells, a detailed inventory of their ability to produce cytokines and degranulate has not been performed. Specifically, fetal liver and spleen NK cell functions have not been directly compared, nor have CXCR6^+^ NK cells in fetal tissue been directly examined in terms of their ability to produce cytokines or degranulate in response to stimuli. We therefore used chromium release assays to determine if fetal liver and spleen NK cells were capable of killing MHC class I deficient K562 target cells and if these NK cells are responsive to IL-2. We had limited available tissue to perform these assays (fetal liver, *n* = *4*; fetal spleen *n* = *2*). Bulk lymphocytes were used since magnetic separation of NK cells would have greatly depleted cell numbers. Fetal liver and spleen NK cells are capable of extremely low levels of lysis of K562 targets over background (<2%). A characteristic effector to target ratio curve can be seen when diluting fetal liver (blue) or spleen effector cells (green). This killing was boosted slightly with the addition of 1,000 units/mL recombinant human IL-2, although it was not statistically significant (squares, Figure 5A). PB NK cells were run with each experiment as a positive control (black). We were only able to perform a maximum effector to target ratio of 25 to 1 for fetal bulk spleen cells, since cell numbers were limited. Fetal spleen NK cells appear to possess more robust cytotoxic activity, relatively speaking, than the fetal liver cells. For example, at the 25:1 effector to target ratio, compare percent specific lysis for fetal liver vs. fetal spleen NK cells with and without IL-2. We also performed antibody-dependent cytotoxicity assays (ADCC) with Raji as targets in the presence or absence of rituximab (Figure 5A). Although cytotoxic activity was extremely low, especially compared to adult PB NK cells, we were able to see a characteristic curve for killing in both fetal liver and spleen Table S3. Overall, the level of cytotoxicity achieved by fetal liver and spleen NK cells is negligible, and not unexpected.

In order to more fully examine the cytotoxic capability of fetal liver and spleen NK cells, we performed degranulation assays. Bulk fetal liver and spleen cells were incubated with CFSE-labeled K562 at a 1:1 ratio or stimulated with PMA/ionomycin (P/I) for 4 h as described in Materials and Methods. Fluorophore-labeled anti-CD107a antibody was added during the incubation to study degranulation and intracellular flow cytometry was performed to detect levels of IFNγ, TNFα, perforin, and granzyme B. We first examined the expression of these markers in total NK cells from fetal liver, fetal spleen, and PB. IFNγ and TNFα expression is virtually absent in all three tissues at baseline (unstimulated) (Figure 5B, top row, “unstim”). Treatment with P/I for 4 h results in a significant increase in IFNγ and TNFα in total NK cells from all three tissues. The percentages of IFNγ-producing NK cells are higher in PB and fetal liver than in fetal spleen (*p* < 0.0001) (Figure 5B). IFNγ and TNFα are only slightly upregulated in PB NK cells, and not at all in fetal liver and spleen NK cells in response to a 1:1 ratio of K562 target cells. Perforin and granzymes are important mediators of NK cell killing. PB NK cells are known to contain high levels of perforin and granzyme B, which are released during NK cell mediated lysis of target cells (66). We used PB NK cells as a positive control for perforin and granzyme B expression. Levels of perforin and granzyme B were very high in PB NK cells, but much lower in fetal liver and spleen NK cells (Figure 5B, second row). This is not unexpected and is most likely one of the reasons that fetal liver and spleen NK cells are not efficient killers. Perforin and Granzyme B mean fluorescence intensity (MFI) typically decreased in PB NK cells upon stimulation, but this did not occur consistently in fetal liver and spleen NK cells, perhaps due to differences in developmental age among the samples (data not shown). Fetal spleen NK cells express more granzyme B than fetal liver NK cells (mean 33 vs. 15%, respectively), but this difference is not statistically different. In summary, percent perforin^+^ and granzyme B^+^ NK cells remain the same in all three tissues upon stimulation, while perforin and granzyme B MFI decrease only in PB NK cells indicating more efficient degranulation and release of perforin and granzyme into target cells. The last marker of cytotoxic effector function we interrogated is CD107a. CD107a or lysosomal-associated membrane protein 1 (LAMP-1) is expressed on lysosomal membranes and cannot be detected unless the cell is undergoing the degranulation process of releasing cytotoxic granules containing perforin and granzymes. CD107a expression is negligible in unstimulated PB NK cells, but is induced in response to P/I and K562 target cells. Fetal liver and spleen NK cells express more CD107a at baseline than PB NK cells, and compared with these particular PB NK cell donors actually express higher levels of CD107a after P/I treatment. Conversely incubation with K562 does not induce high levels of CD107a expression in fetal liver and spleen NK cells (Figure 5B, bottom row).

### CXCR6^+^ Fetal Liver and Spleen NK Cells Possess a Unique Functional Profile With Respect to Mediators of NK Cell Effector Function

CXCR6^+^ NK cells in adult liver possess a tissue-resident immature phenotype (CD56^bright^, CD69^+^, CD49e^−^, CD16^lo^, CD57^lo^, KIR^lo^, and NKG2A^+^) (14), Figure 1C). Differences in the ability to secrete cytokines, perforin, and granzyme B expression have been studied in adult liver (14). We investigated differences in cytokine production between CXCR6^−^ and CXCR6^+^ fetal liver and spleen NK cells, and differences in perforin and granzyme B expression upon stimulation (Figures 5C–E). There was little to no IFNγ or TNFα production amongst unstimulated CXCR6^−^ and + NK cell subsets in any tissue (Figure 5B and data not shown). Upon stimulation with P/I, CXCR6^+^ NK cells in the fetal liver and spleen produce more IFNγ than CXCR6^−^ from the same tissue (*p* < 0.01 and *p* < 0.05, respectively) (Figure 5C). This is not the case in PB NK cells where no difference in IFNγ production was seen between the subsets. K562 (1:1 ratio) induces a small amount of IFNγ in CXCR6^−^ NK cells in all three tissues, but there is no difference in IFNγ production between the two subsets (Figure 5C). CXCR6^+^ NK cells from fetal liver produce more TNFα than CXCR6^−^ NK cells (*p* < 0.05) but there is no statistical difference in ability to produce TNFα between the subsets in fetal spleen or PB upon stimulation with P/I, although the trend is toward significance in fetal spleen. K562 induces small amounts of TNFα in fetal liver and spleen NK cells, mostly in the CXCR6^−^ subset (*p* = 0.055 and *p* < 0.05, respectively), which is in agreement with adult liver data (14). There is no difference in the ability of CXCR6^−^ and + NK cells in PB to produce TNFα with either stimulus, although the trend with K562 stimulation is higher TNFα in the CXCR6^−^ subset (Figure 5C).

We next studied baseline perforin expression in CXCR6^−^ and + NK cells and changes in perforin expression following stimulation. In unstimulated NK cells, PB NK cells express high levels of perforin, which is a pore-forming protein known to allow pro-apoptotic proteases like granzyme B to enter target cells (67). There does not seem to be a significant difference in perforin levels between CXCR6^−^ and + NK cells in PB, but the trend is toward lower perforin expression in CXCR6^+^ PB NK cells upon stimulation (Figure 5D). This was not discernable from the total NK cell graph in Figure 5B, and indicates more perforin release in CXCR6^+^ PB NK cells upon P/I stimulation (*p* = 0.053). There was no decrease in perforin in either CXCR6^−^ or + PB NK cells upon stimulation with K562. In fetal liver, CXCR6^+^ NK cells appear to express more perforin at baseline (unstimulated, data not shown) than CXCR6^−^ NK. This relationship holds through both stimulation conditions, with CXCR6^+^ NK cells always expressing more perforin, but is not statistically significant. In fetal spleen, there is no statistical difference in perforin expression between CXCR6^−^ and CXCR6^+^ NK cells. CXCR6^+^ NK cells in adult liver are more likely to degranulate (CD107a expression) than their CXCR6^−^ NK cells counterparts after stimulation with P/I (14). We found this to be the case as well in both fetal liver and spleen NK cells (Figure 5D), although the difference is not significant in fetal liver. Degranulation was very low in response to K562, with no significant difference between the subsets in fetal liver and spleen or adult PB (Figure 5D).

Granzyme B is a serine protease located in the cytotoxic granules of NK cells. In all tissues, CXCR6^+^ NK cells express significantly less granzyme B at baseline (unstim) and under both stimulation conditions than CXCR6^−^ NK cells (Figure 5E). Granzyme B expression levels (%^+^NK) do not change significantly following stimulation (total NK cell graph, Figure 5B). Granzyme B MFI typically decreases following PB NK cell stimulation signifying release into the target cell. We saw no significant decrease in granzyme B MFI in fetal liver or spleen NK cells following stimulation (data not shown). Taken together, CXCR6^+^ NK cells in fetal liver and spleen are generally capable of secreting more IFNγ, TNFα, and CD107a than their CXCR6^−^ counterparts.

## Discussion

There has been a recent surge of interest in tissue-resident NK cells from various organs throughout the body including, spleen, liver, tonsil, lymph node, endometrium, and decidua (11, 14, 18, 19, 28–30, 38, 68–70). Tissue resident CD56^bright^ NK cells make up the majority of NK cells in the body, but the developmental origin and specific function of these cells independent of their CD56^bright^ PB counterparts is not completely clear, as the most frequently studied NK cell is PB rather than tissue derived. The distinction of CD56 dim vs. bright NK cells, and their designations as mature (dim) vs. immature (bright) is thereby based predominantly on the evaluation of human PBMC-derived NK cells rather than the study of tissue resident NK. In light of new data, it may be time to reconsider whether this type of thinking is overly simplistic. Indeed, some studies have proposed that CD56 dim and bright NK cells may be separate lineages of NK cells (71–75). Indeed, a recent study demonstrated that there are up to 30,000 subsets of NK cells in PB of health human adult donors, leaving us to wonder how many different NK cell subsets or lineages may reside in human tissues (72).

Tissue resident NK cells possess a unique phenotypic and functional profile indicating a capacity for a unique role in human immunity (37). Adult human liver NK cells are CXCR6^+^, CD69^+^, CD49e^−^, and EOMES^+^. CXCR6 was originally identified as a receptor for Human Immunodeficiency Virus (HIV) (76), and has more recently been implicated in NK cell memory in murine liver NK cells (17). Both hepatic and splenic NK cells have also been implicated in NK cell memory in rhesus macaques (25). The prevalence of CXCR6^+^ NK cells in secondary lymphoid organs, particularly liver, may be partially explained by the high expression of the ligand for CXCR6, CXCL16, in liver sinusoid epithelium, which is thought to retain CXCR6^+^ NK cells in liver and provide them with a survival signal (17). Whether CXCR6 is already expressed on fetal NK cells, and how fetal CXCR6^+^ NK cells differ from their CXCR6^−^ counterparts in fetal liver and spleen, and in comparison to adult liver and spleen, has not been explored. An obvious limitation of our study and others examining fetal NK cells is the small number of samples available at each gestational age. Nevertheless, we performed a direct phenotypic and functional comparison of total, CXCR6^−^, and CXCR6^+^ NK cells from fetal liver and spleen and compared them to adult PB, liver, and spleen NK cells. Understanding the phenotypic and functional profiles of fetal NK cells may inform the utility of these subsets as capable responders to maternal or *in utero* vaccination.

Overall, fetal hepatic and splenic NK cells are classically immature, resemble each other more than PB NK cells and are capable of low-level cytotoxicity and ADCC. In addition, fetal liver and spleen NK cells phenotypically resemble their adult tissue counterparts as early as 17 weeks in terms of tissue-residency markers, including CXCR6 and CD69 (Figure S11A). In general, NK cells in fetal liver and spleen are immature with respect to CD16, CD57, KIR, DNAM-1, CD62L, NKG2A, CD94, and CD27/CD11b expression. Activation receptors NKG2D and NKp46 mirror each other in fetal liver and spleen NK, while CD69 is more highly expressed on fetal liver and spleen NK cells than PB NK cells, as expected. Both fetal liver and spleen NK cell receptor repertoires are fundamentally in place during the gestational time frame we studied (17–23 weeks), with the exception of some maturation and migration markers (CD57, KIR, and CD62L). Similar results have been obtained for cord blood NK cells suggesting that vaccine strategies could potentially be geared toward the newborn NK cell repertoire (71), or perhaps even *in utero*.

For example, newborns are routinely vaccinated with *Mycobacterium bovis* bacillus Calmette-Guerin (BCG) in many parts of the world (77). Despite this, the rate of Tuberculosis (TB) in neonates remains unacceptably high (78). IFNγ production following the birth of BCG-vaccinated infants can be stimulated in cord blood *ex vivo* with BCG. NK cells are the main source of this IFNγ, which is crucial for clearing TB infection. Hence, this early response in neonates is not a T cell response, but an innate immune (NK) cell response (77). Further, cord blood NK cells can be directly stimulated with BCG to produce IFNγ, in addition to being stimulated by the IL-12 produced by dendritic cells (DCs) in response to BCG (79). The fact that this IFNγ production was found to be mediated by CD16^lo^ NK cells in a TLR-2 dependent manner is highly relevant to the current study since tissue-resident CXCR6^+^ NK cells in the fetal liver and spleen are classically CD16^lo^ and are therefore potentially equipped to respond to vaccination or specific viral threats during gestation by producing IFNγ.

Congenital CMV infection is of major concern during pregnancy due to possible neurological defects, including hearing impairment, mental retardation, and cerebral palsy, particularly if newborns are infected during the first trimester of gestation (80). In a manner similar to adults, NKG2C^+^ NK cells are expanded in young children who had been infected with CMV *in utero* and the percent expanded NK cells is higher in symptomatic children (81, 82). Moreover, a 3 months old Severe Combined Immunodeficiency (SCID) patient who had no T cells, but had B and NK cells spontaneously resolved a gastroenteritis caused by CMV without anti-viral treatment, demonstrating an important role of NK cells in the control of CMV infection. There is evidence that T cell exhaustion may limit anti-viral T cell responses early in life, which would make NK cell responses even more critical in controlling infection (81).

Understanding the capabilities of the fetal innate immune system, specifically NK cells, and how these NK cells may further influence the adaptive response could potentially aid in the design of new and better vaccination strategies for infectious diseases including TB (77) and CMV, and perhaps also autoimmune diseases or allergies.

Several notable characteristics make fetal spleen NK cells appear more mature and more capable of cytotoxic effector function than fetal liver NK cells. One reason for this may be the higher number of circulating NK cells from the periphery in this organ as compared to fetal liver. Adult spleen NK cells also favor the mature end of the spectrum for tissue-resident NK cells in that they are higher in KIR, and although not statistically significant, higher in Tbet^+^EOMES^−^ NK cells than their adult liver counterparts. Direct phenotypic comparison of CXCR6^−^ and + NK cells in adult liver and spleen is beyond the scope of this manuscript.

A distinct CXCR6^+^ NK cell phenotype has been described for adult liver (14, 29) and is actually also readily apparent in PB, even more than in fetal liver or spleen, most likely due to the lack of maturation markers in fetal NK cells, which is where many of the differences occur (i.e., CD16, CD57, and KIR). Despite this, the beginnings of a discrete CXCR6^+^ NK cell subset in fetal liver and spleen can be evidenced by differences in the expression of numerous differentiation markers. In terms of the ability of these NK cells to migrate, CXCR6^+^ fetal NK cells appear to remain in the secondary lymphoid organs, while CXCR6^+^ PB NK cells seem poised to migrate to lymph nodes or sites of inflammation.

The tissue-residency marker CD69 is expressed at very low levels on total PB NK cells. Previous publications have asserted that CD69 is completely absent from PB NK cells (18). While this is indeed the case when analyzing total NK cells (Figure 1B), CD69 is significantly higher on CXCR6^+^ NK cells in PB than on their CXCR6^−^ counterparts (Figure 2A). This is not readily apparent when analyzing total NK cells since the majority of these cells are CXCR6^−^. This raises the question of whether CXCR6^+^ NK cells in the periphery originate in secondary lymphoid organs like liver and spleen or are induced to express CD69 during an immunologic event in the periphery and then eventually home to liver to reside as memory NK cells. Circulating EOMES^lo^ NK cells are precursors of their liver resident counterparts and they can be induced to become EOMES^hi^ NK cells and express markers that facilitate liver retention (11). Furthermore, based on tracking liver NK cells from liver transplants with mismatched HLA recipients and donors, EOMES^hi^ liver NK cells are unable to leave the liver even after 13 years. Higher CXCR6^+^ NK cells in fetal liver and spleen allows for retention of these NK cells in their respective secondary lymphoid organs, where they help maintain an immuno-tolerant environment.

Absence of CD49e is another recently discovered candidate for delineating tissue-residency in the liver (30). CD49e (VLA-5) mediates adhesion of NK cells to fibronectin, and may be important for NK cells to interact with the extracellular matrix (83). Interestingly, CD49e is higher in fetal NK cells and is drastically reduced or completely lost in the CXCR6^+^ NK cell subset of both organs in adult tissue. This indicates that CD49e may be lost as NK cells in these organs mature. It is significant that CD49e^−^ liver NK cells highly resemble CXCR6^+^ NK cells in that they are CD69^+^, CD94^+^, CD27^+^, and lower in CD62L (30). It is possible that the co-expression of CXCR6 with low CD49e may demarcate a tissue-resident NK cell population in adult spleen, but this has yet to be thoroughly explored.

Notably, DNAM-1 is also differentially expressed on CXCR6^+^ NK cells in fetal spleen and may represent an alternate maturation pathway in this organ. DNAM-1^+^ NK cells produce higher levels of cytokines and can differentiate into DNAM-1^−^ NK cells (50). Hence, DNAM-1 expression may distinguish two different functional NK cell subsets, which may also be demarcated by CXCR6 expression in fetal spleen (Figure 2A).

Functionally, CXCR6^+^ fetal liver and spleen NK cells produce more IFNγ and TNFα and degranulate more (in fetal spleen) than their CXCR6^−^ NK cell counterparts when stimulated with PMA/ionomycin. This is the opposite of what is published in adult liver, where CXCR6^−^ NK cells produce more IFNγ and TNFα than CXCR6^+^ NK cells (14), however, the concentrations of PMA and ionomycin used differ between studies. CXCR6^+^ NK cells do, however, degranulate more than CXCR6^−^ NK cells in adult liver (14) and fetal spleen (Figure 5G). Total fetal liver and spleen NK cells also degranulate well in response to a combination of IL-12 and IL-18 (32), while K562 is not as potent a stimulus for fetal liver and spleen NK cells as PMA/ionomycin. This is not surprising since fetal lung NK cells also respond poorly to other HLA class I-negative targets (32). In light of the functional data, fetal NK cells are highly capable of activation, and CXCR6^+^ NK cells show a unique activation profile compared to CXCR6^−^ NK cells in fetal liver and spleen, which is not seen in PB NK cells (Figure 5).

In conclusion, CXCR6^+^ NK cells constitute the majority of fetal liver NK cells and represent a large proportion of the NK cells in fetal spleen. Fetal NK cells in liver and spleen have already acquired CXCR6 and many of the phenotypic characteristics of their adult counterparts that help define them as tissue resident as early as week 17. NK cells from newborns (cord blood) also have a phenotypically complete repertoire, as mentioned above. “There is only a slight lack of functional readiness to overcome when presented with the right stimulus” (71). Fetal liver and spleen NK cells can be activated given the appropriate stimulus, and CXCR6^+^ NK cells exhibit a different functional profile from their CXCR6^−^ counterparts, which may be indicative of a specialized function, which may include IFNγ/TNFα secretion and degranulation. Interestingly, CD8^+^ T cells from newborns can also mount a significant immune response as evidenced by the expansion of a subset of CD8^+^ T cells in response to HCMV infection (84), and recently tissue resident memory CD8^+^ T cells have been described in adult human liver. These CD8^+^ T cells closely resemble their NK cell counterparts (CXCR6^+^ CD69^+^ Tbet^lo^) (85). Taken together, these results provide insight as to when human NK cells develop specialized tissue-resident phenotypes and functions, and how they can potentially be recruited for use *in utero* and in newborn or maternal vaccine development.

## Data Availability

All datasets generated for this study are included in the manuscript and/or the supplementary files.

## Ethics Statement

All human peripheral blood and adult spleen samples were obtained by written informed consent and the protocol was approved for use by the National Institutes of Health (NIH) and Baylor College of Medicine Institutional Review Boards for the Protection of Human Subjects, and in accordance with the Declaration of Helsinki.

## Author Contributions

SP directed the research and designed experiments. LA, RN, and KA-P performed the experiments. LA, LB, and SP analyzed the data, and LA and SP wrote the manuscript. All authors contributed to manuscript revision, read, and approved the submitted version.

### Conflict of Interest Statement

The authors declare that the research was conducted in the absence of any commercial or financial relationships that could be construed as a potential conflict of interest.
