# Supplementary figures and images for: CXCR6+ NK Cells in Human Fetal Liver and Spleen Possess Unique Phenotypic and Functional Capabilities
Source: Front Immunol. 2019 Mar 19;10:469. doi: 10.3389/fimmu.2019.00469 (PMC6433986; doi:10.3389/fimmu.2019.00469)

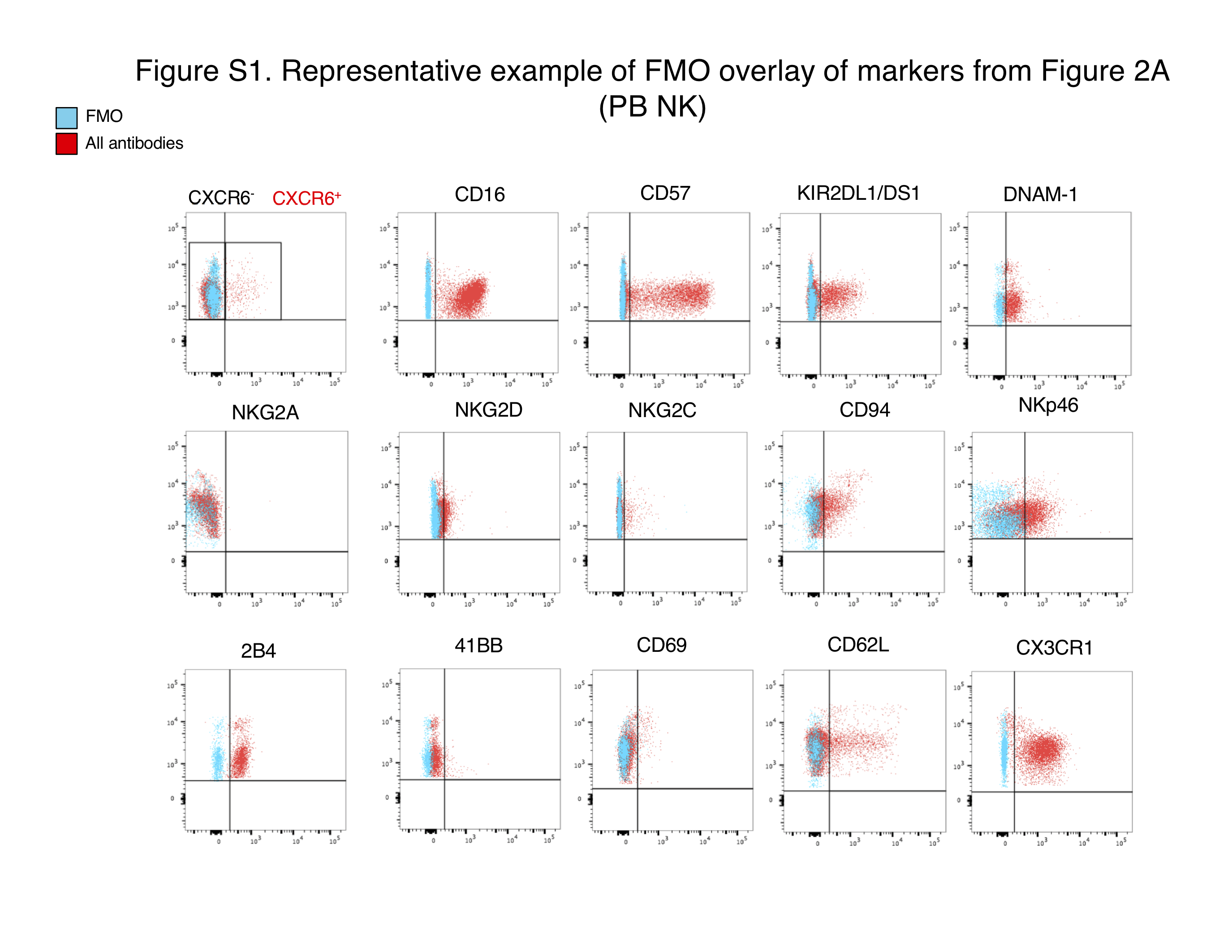

Supplement: Supplementary file 1 [file Image_1.tiff]

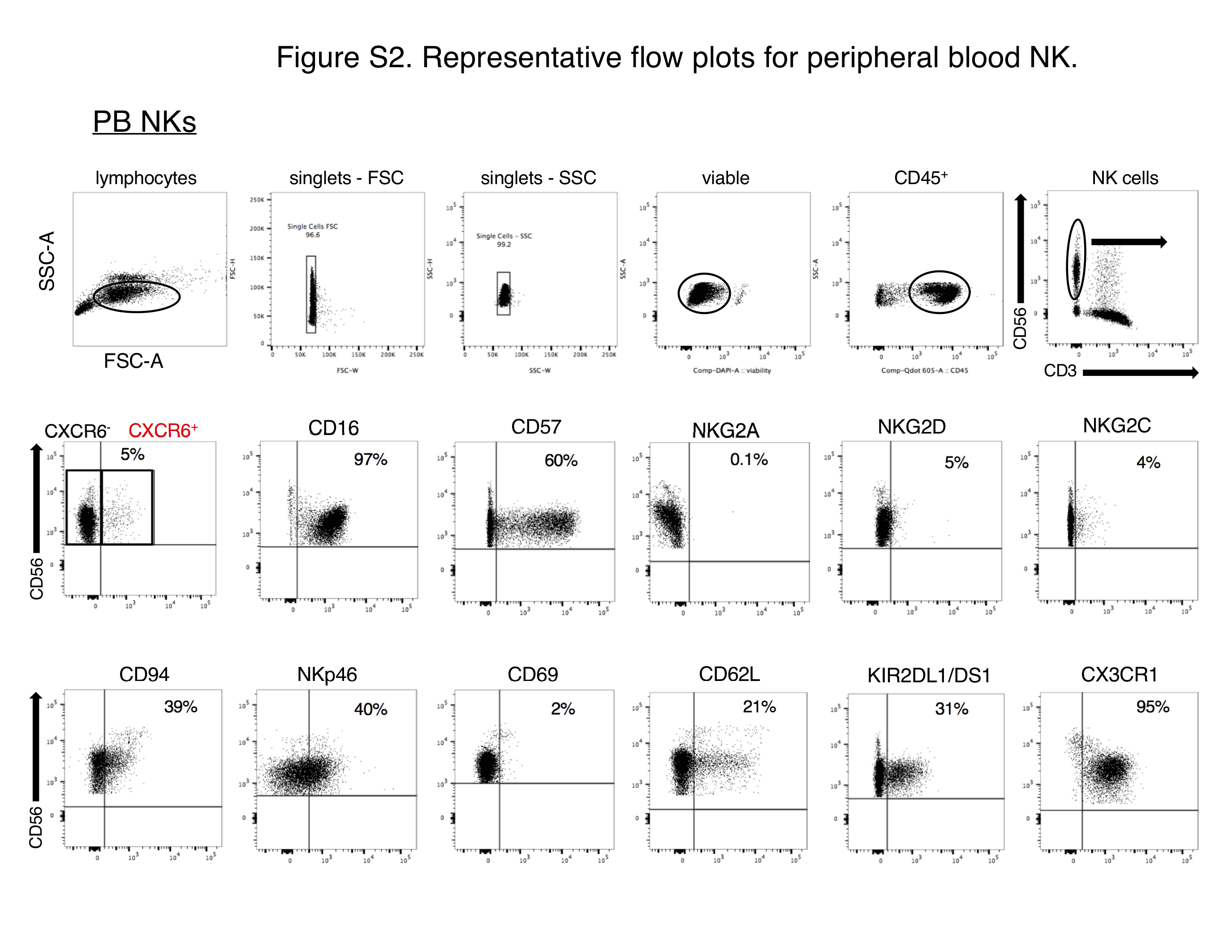

Supplement: Supplementary file 2 [file Image_2.tiff]

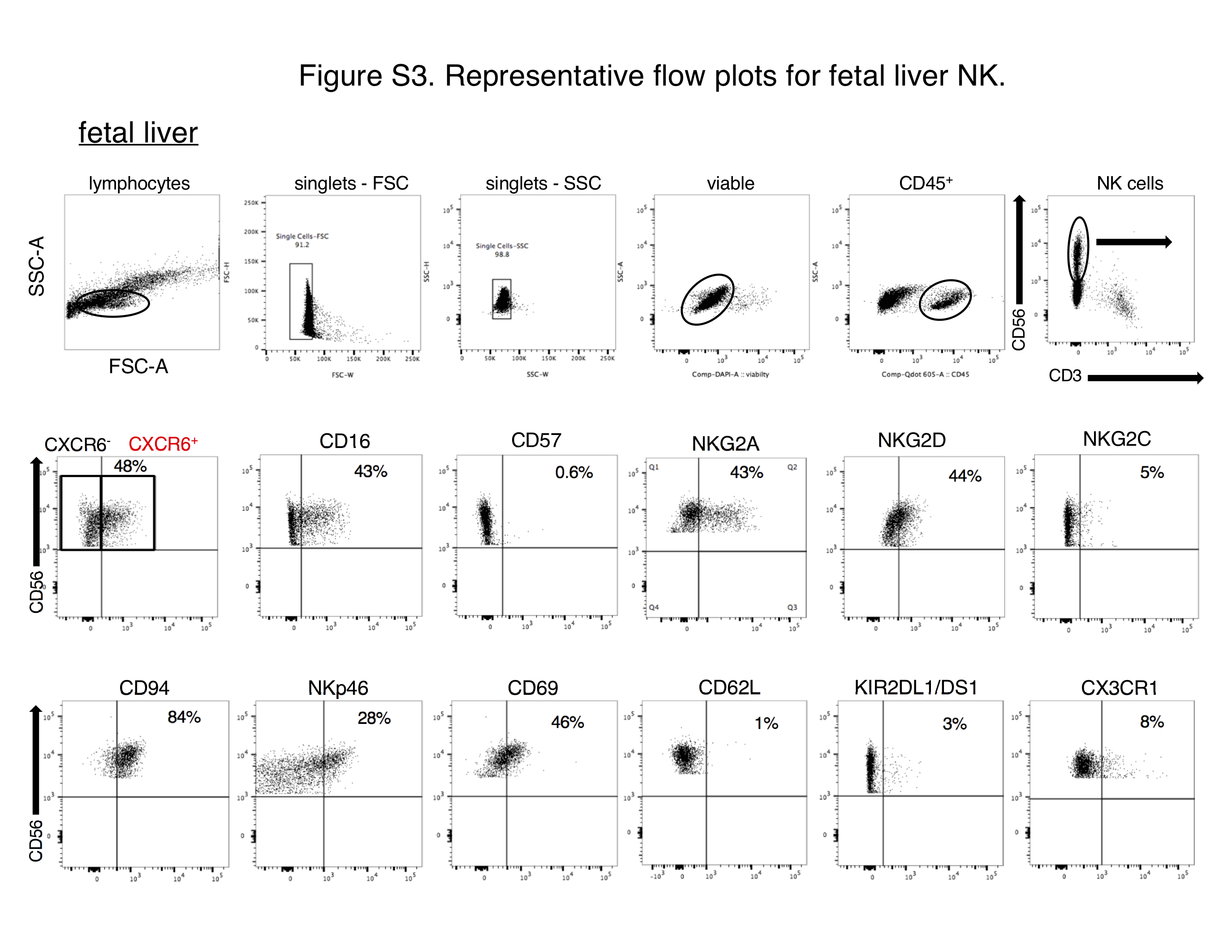

Supplement: Supplementary file 3 [file Image_3.tiff]

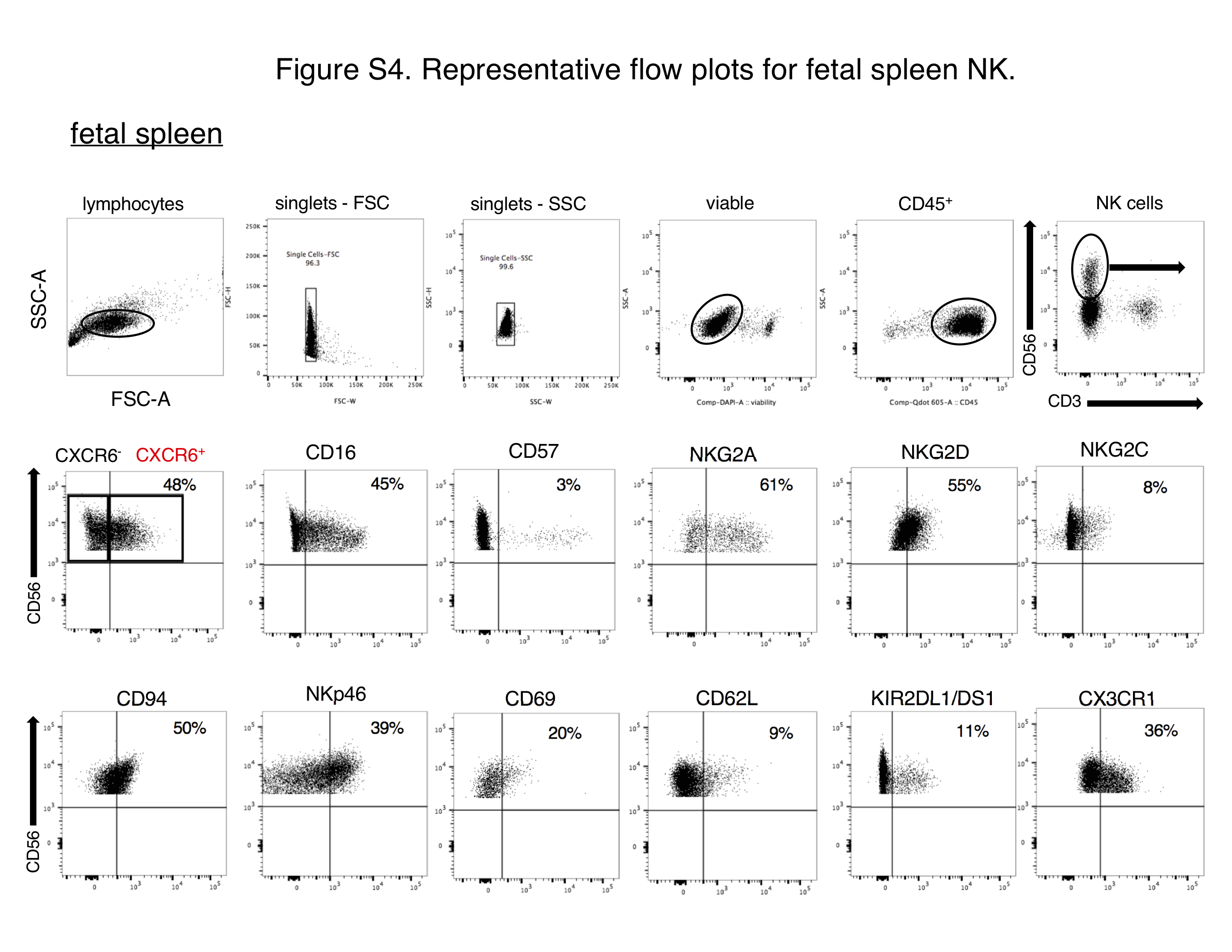

Supplement: Supplementary file 4 [file Image_4.tiff]

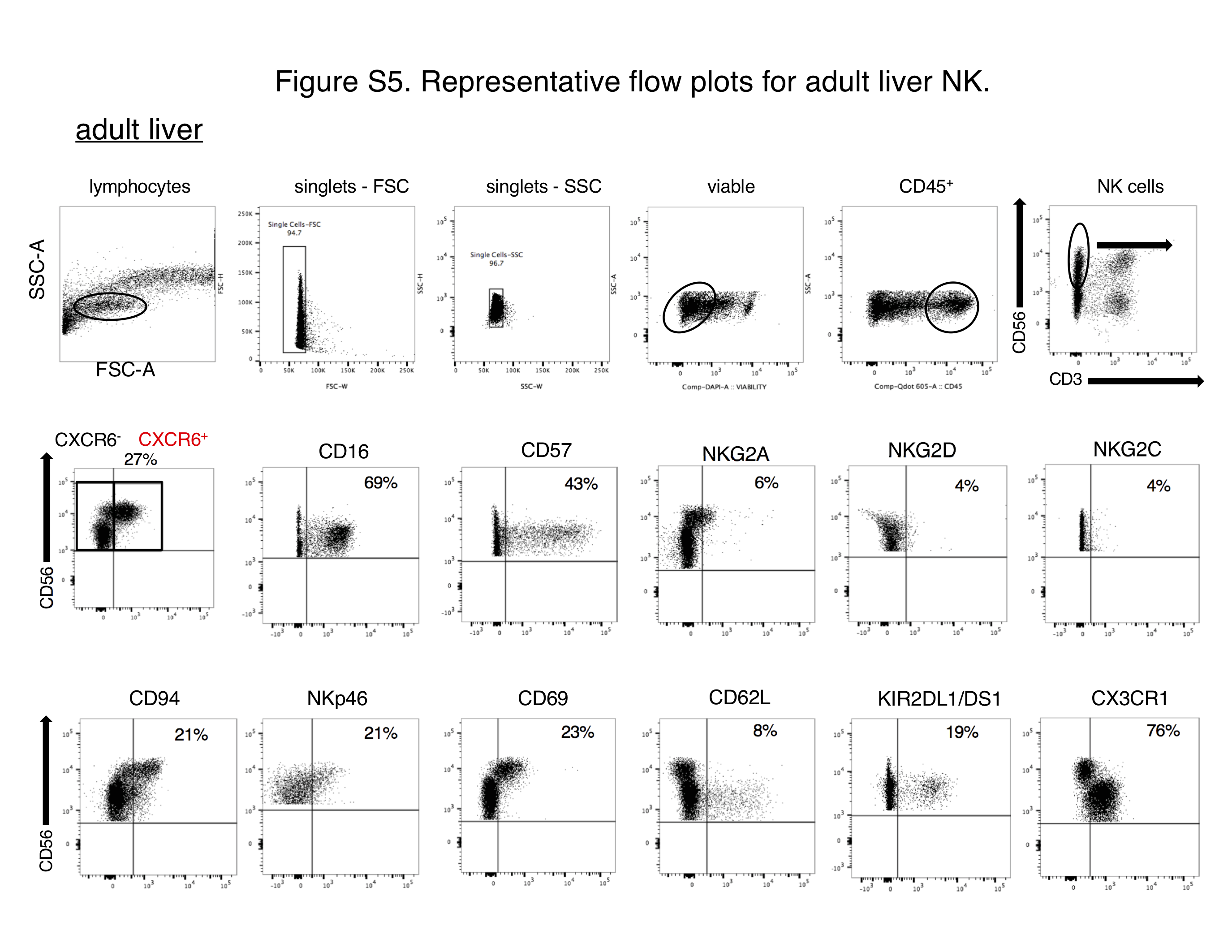

Supplement: Supplementary file 5 [file Image_5.tiff]

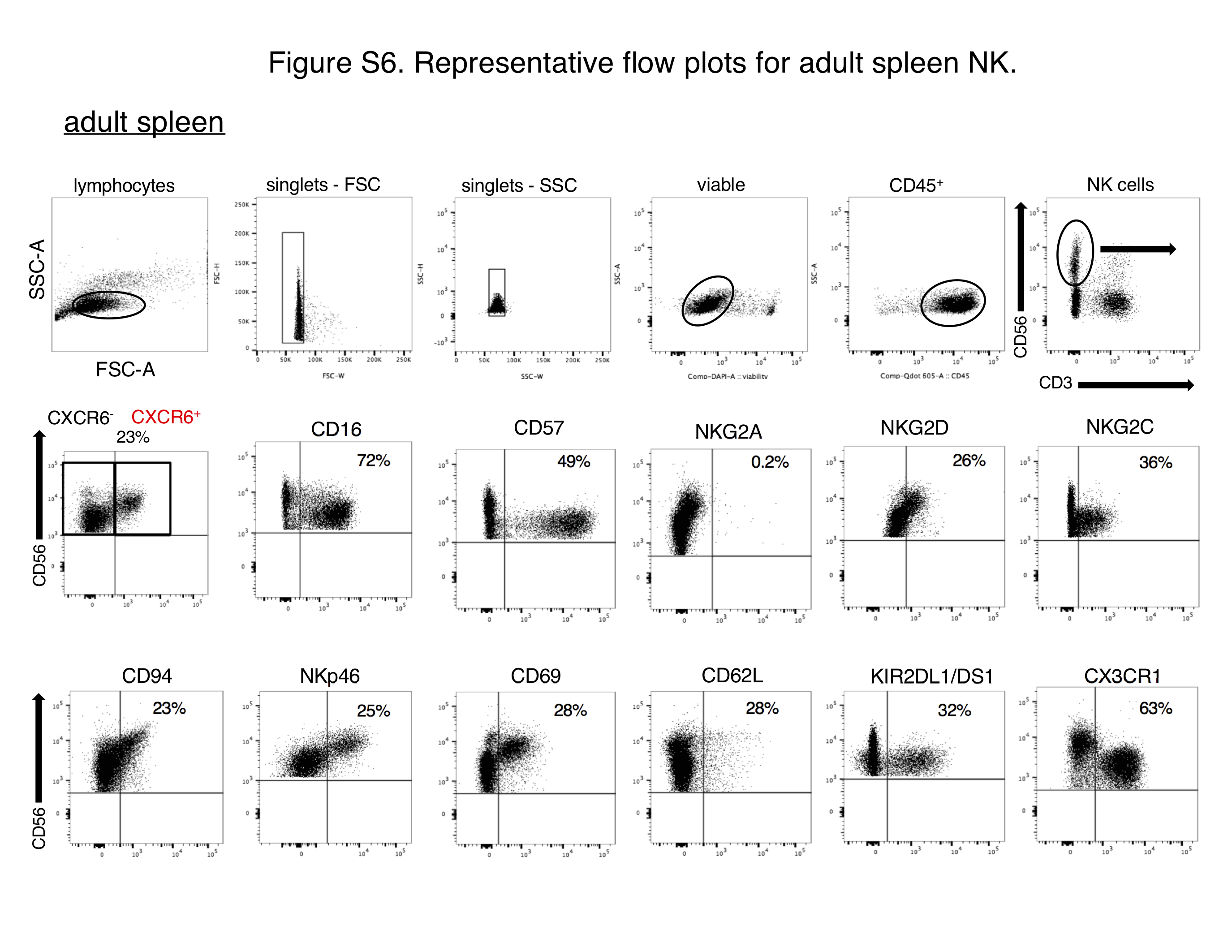

Supplement: Supplementary file 6 [file Image_6.tiff]

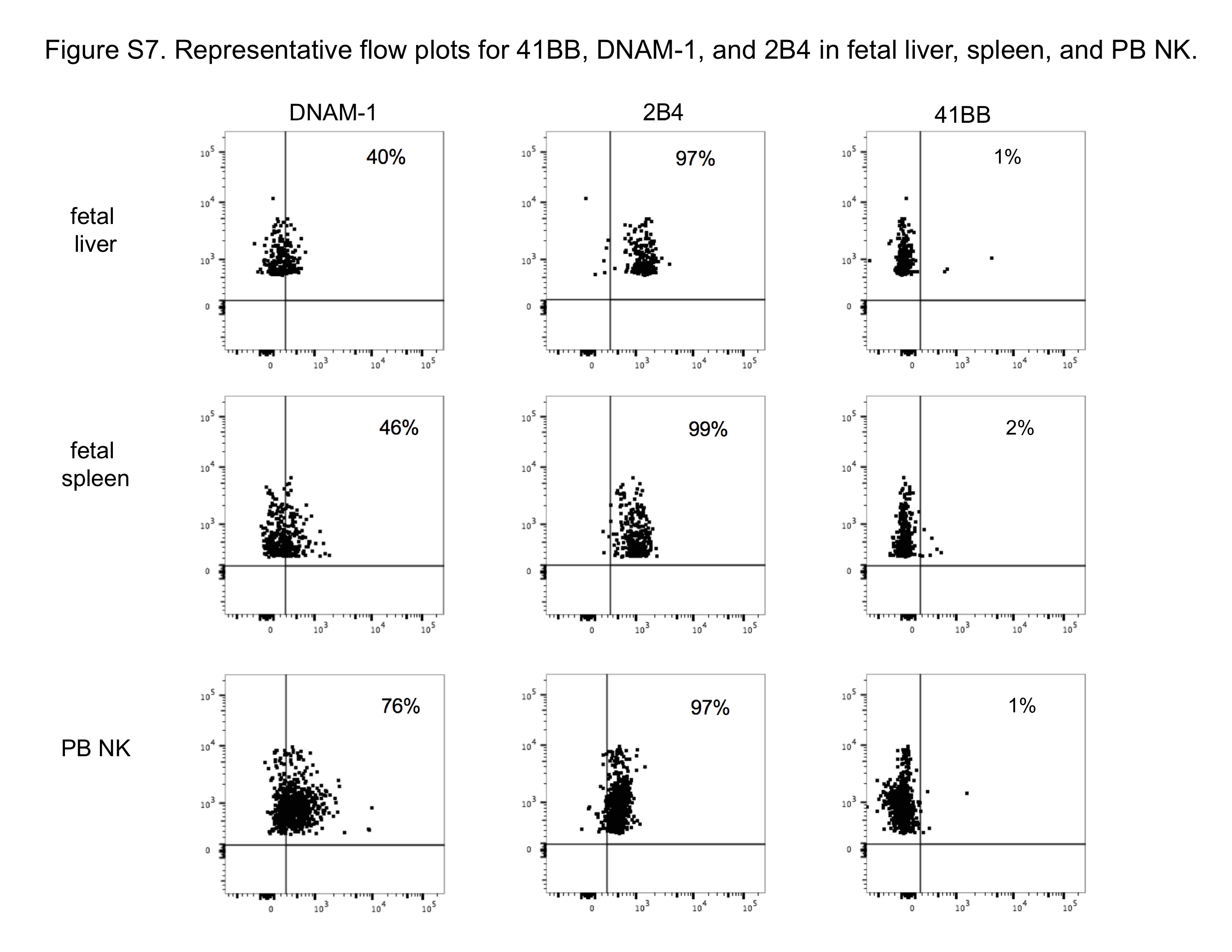

Supplement: Supplementary file 7 [file Image_7.tiff]

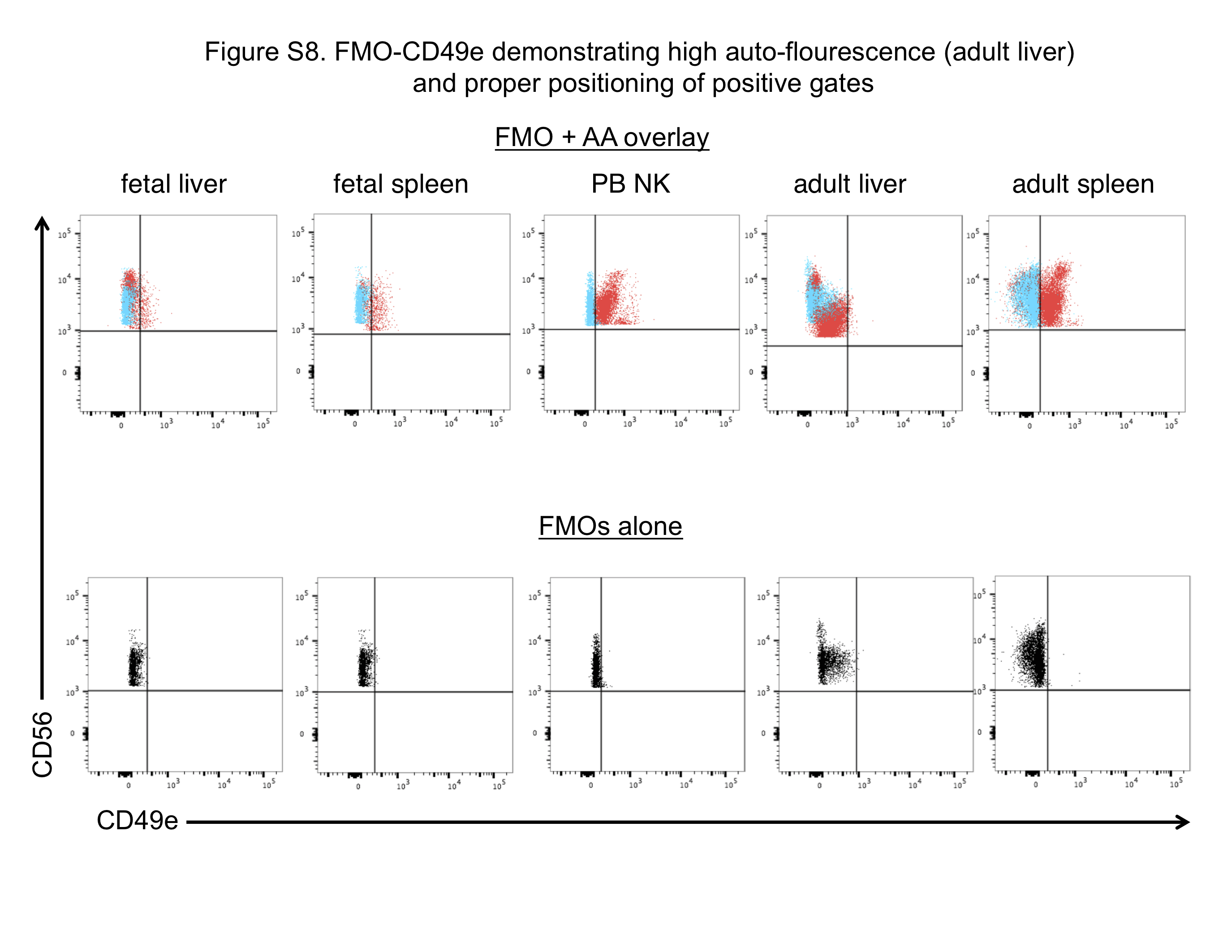

Supplement: Supplementary file 8 [file Image_8.tiff]

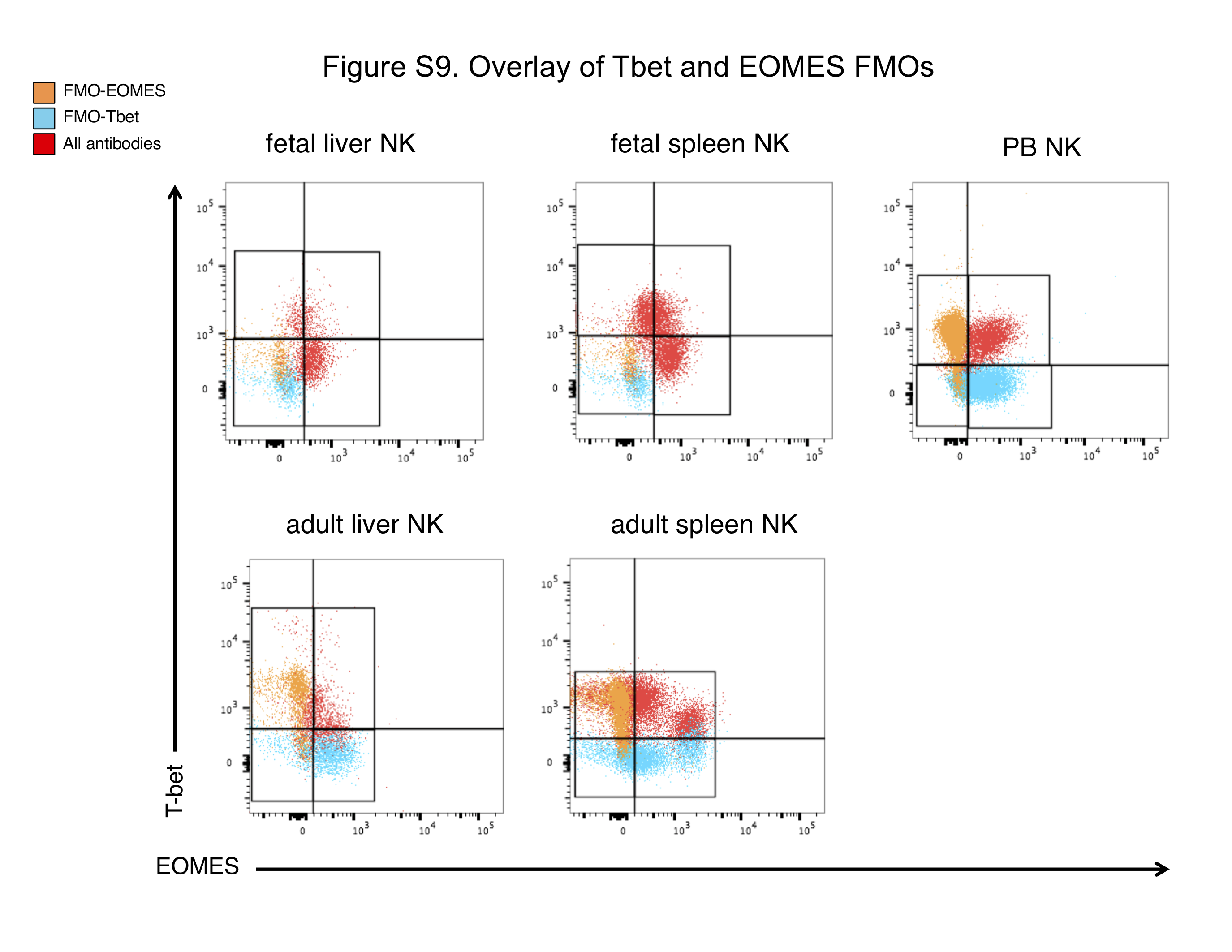

Supplement: Supplementary file 9 [file Image_9.tiff]

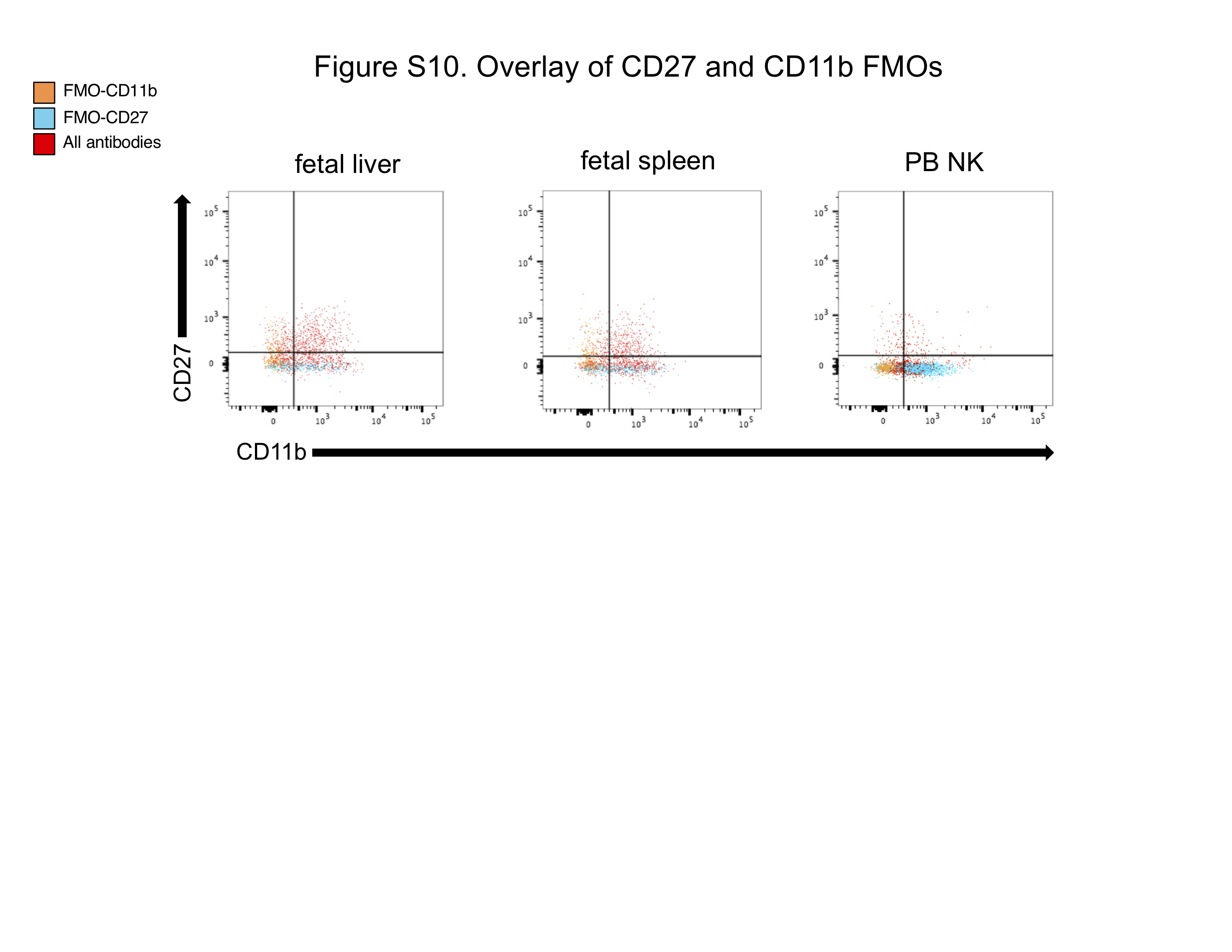

Supplement: Supplementary file 10 [file Image_10.tiff]
