# Supplementary material for: CXCR6+ NK Cells in Human Fetal Liver and Spleen Possess Unique Phenotypic and Functional Capabilities
Source: Front Immunol. 2019 Mar 19;10:469. doi: 10.3389/fimmu.2019.00469 (PMC6433986; doi:10.3389/fimmu.2019.00469)

Figure S11A. Expression pattern of NK cell markers as a function of gestational age in fetal liver and spleen NK cells. Maturation markers CXCR6, CD16, CD57, and KIR2DL1-DS1 were analyzed. Percent positive of total NK cells is shown. Fetal liver NK (blue); fetal spleen NK (green). Gestational ages ranged from 17 to 23 weeks. *n = 1 -3*. All data points are shown.

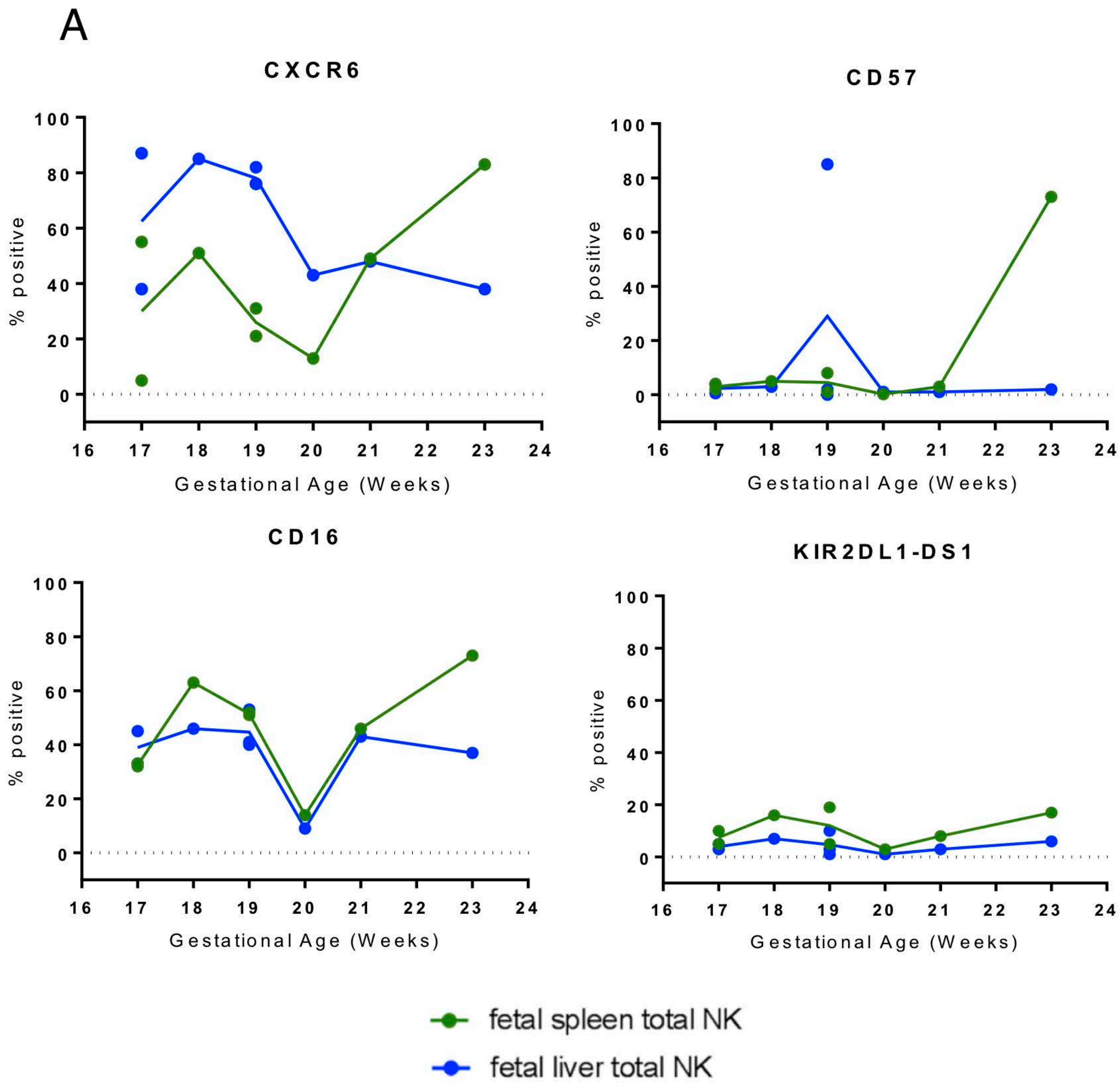

Figure S11B. Comparison of NKG2A, NKG2D, CD94, and natural cytotoxicity receptor NKp46 in total fetal liver and spleen NK cells as a function of gestational age.

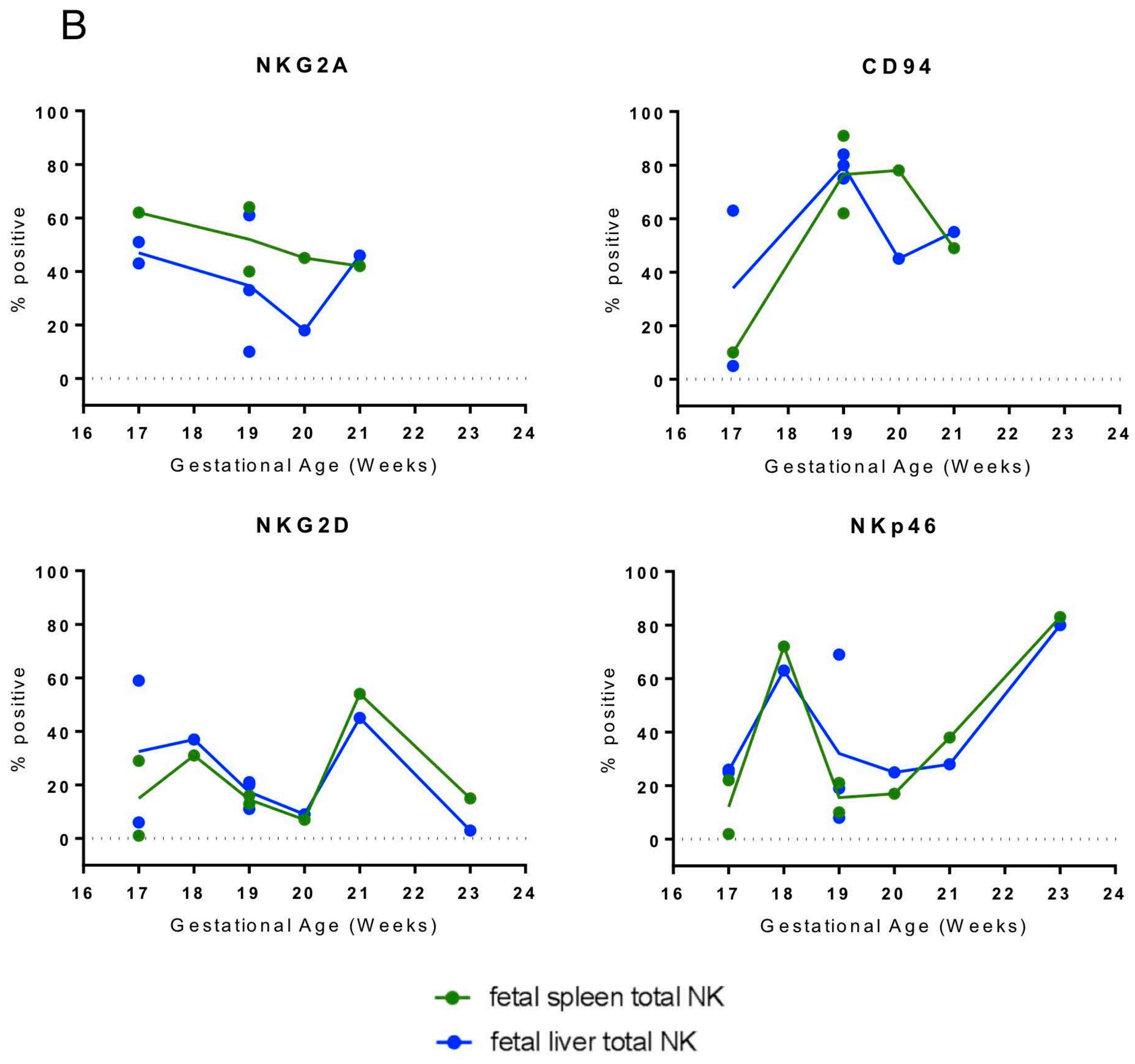

Figure S11C. Tissue-specific (CD49e, CD69), and migration/homing receptors (CD62L, CX3CR1) as a function of gestational age in fetal liver and spleen NK cells.

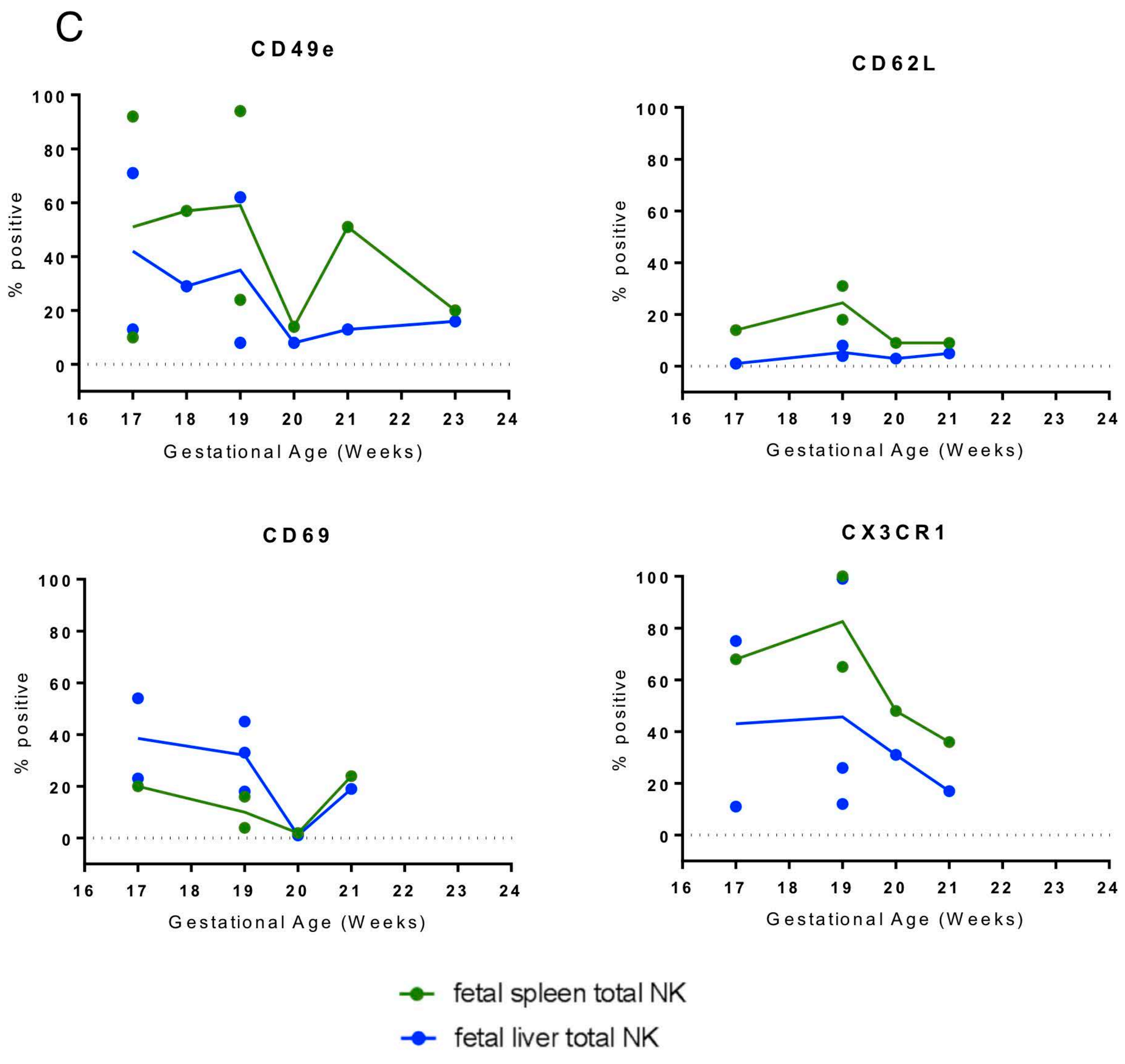

Supplement: Supplementary file 11 [file Image_11.pdf]
