# Supplementary material for: CXCR6+ NK Cells in Human Fetal Liver and Spleen Possess Unique Phenotypic and Functional Capabilities
Source: Front Immunol. 2019 Mar 19;10:469. doi: 10.3389/fimmu.2019.00469 (PMC6433986; doi:10.3389/fimmu.2019.00469)
Supplement: Supplementary file 12 [file Table_1.pdf]

## Supplemental Table 1. Antibodies used for multi-parametric flow cytometry

| Marker | Fluorophore | clone | Company |
|---|---|---|---|
| CD3 | BV785 | OKT3 | Biolegend |
| CD45 | BV605 | HI30 | Biolegend |
| CD16 | APC/Cy7 | B73.1 | Biolegend |
| CXCR6 | AF647 | KO41E5 | Biolegend |
| CD56 | BV421 | HCD56 | Biolegend |
| CD57 | PE-TX Red | HNK-1 | Biolegend |
| CD49e | FITC | NKI-SAM1 | Biolegend |
| NKG2C | PE | 134591 | R & D Systems |
| NKG2D | BV510 | 1D11 | Biolegend |
| NKp46 | BV650 | 9E2/NKp46 | BD Biosciences |
| KIR2DL1-DS1 | PE/Cy7 | 11PB6 | Miltenyi |
| Tbet | BV711 | 4B10 | Biolegend |
| EOMES | PerCP-eFluor 710 | WD1928 | eBioscience |
| NKG2A | APC | 131411 | R & D Systems |
| CD94 | PerCP/Cy5.5 | DX22 | Biolegend |
| CD69 | BV650 | FN50 | Biolegend |
| CD62L | PE/Cy5 | DREG-56 | Biolegend |
| CX3CR1 | PE | 2A9-1 | Biolegend |
| 41BB | AF700 | 4B4-1 | BD Biosciences |
| DNAM-1 | BV711 | DX11 | BD Biosciences |
| 2B4 | PerCP/Cy5.5 | C1.7 | Biolegend |
| CD27 | APC/Cy7 | 0323 | Biolegend |
| CD11b | PE/Cy5 | ICRF44 | Biolegend |
| IFNg | AF700 | 4S.B3 | Biolegend |
| TNFa | PE/Cy7 | Mab11 | Biolegend |
| Perforin | PE | dG9 | Biolegend |
| Granzyme B | BV421 | GB11 | Biolegend |
| CD107a | BV786 | H4A3 | BD Biosciences |

## Supplemental Table 2. Gestational ages of fetal tissue

| Sample # | Gestational age |
|---|---|
| FL11 | 17 weeks |
| FL12 | 17 weeks |
| FL10 | 18 weeks |
| FL4 | 19 weeks |
| FL8 | 19 weeks |
| FL9 | 19 weeks |
| FL3 | 20 weeks |
| FL6 | 21 weeks |
| FL14 | 23 weeks |

Supplemental Table 3. Comparison of cytotoxicity and ADCC effector function in Fetal liver and spleen NK compared to PB NK.

| Organs Compared | Cytotoxicity +/- IL-2 | | | | | | ADCC +/- rit | | | | | | Statistics |
|---|---|---|---|---|---|---|---|---|---|---|---|---|---|
| | 3.13 | 6.25 | 12.5 | 25 | 50 | 100 | 3.13 | 6.25 | 12.5 | 25 | 50 | 100 | |
| Within Fetal Liver | n.s. | n.s. | n.s. | n.s. | n.s. | n.s. | n.s. | n.s. | n.s. | n.s. | n.s. | n.s. | Two-way ANOVA with Tukey's Multiple Comparison Test |
| Within Fetal Spleen | n.s. | n.s. | n.s. | n.s. | | | n.s. | n.s. | n.s. | n.s. | | | |
| Within PB | n.s. | n.s. | n.s. | n.s. | * | * | n.s. | n.s. | *** | **** | **** | **** | |
